# Interaction With the Lipid Membrane Influences Fentanyl Pharmacology

**DOI:** 10.3389/adar.2022.10280

**Published:** 2022-03-21

**Authors:** Katy J. Sutcliffe, Robin A Corey, Norah Alhosan, Damiana Cavallo, Sam Groom, Marina Santiago, Chris Bailey, Steven J. Charlton, Richard B. Sessions, Graeme Henderson, Eamonn Kelly

**Affiliations:** ^1^ School of Physiology, Pharmacology and Neuroscience, Faculty of Life Sciences, University of Bristol, Bristol, United Kingdom; ^2^ Department of Biochemistry, Medical Sciences Division, University of Oxford, Oxford, United Kingdom; ^3^ Department of Pharmacy and Pharmacology, Faculty of Science, University of Bath, Bath, United Kingdom; ^4^ Macquarie Medical School, Macquarie University, Sydney, NSW, Australia; ^5^ Physiology, Pharmacology and Neuroscience, School of Life Sciences, University of Nottingham, Nottingham, United Kingdom; ^6^ School of Biochemistry, Faculty of Life Sciences, University of Bristol, Bristol, United Kingdom

**Keywords:** opioid, fentanyl, lipid, molecular dynamics, binding

## Abstract

Overdose deaths from fentanyl have reached epidemic proportions in the USA and are increasing worldwide. Fentanyl is a potent opioid agonist that is less well reversed by naloxone than morphine. Due to fentanyl’s high lipophilicity and elongated structure we hypothesised that its unusual pharmacology may be explained by its interactions with the lipid membrane on route to binding to the µ-opioid receptor (MOPr). Through coarse-grained molecular dynamics simulations, electrophysiological recordings and cell signalling assays, we determined how fentanyl and morphine access the orthosteric pocket of MOPr. Morphine accesses MOPr via the aqueous pathway; first binding to an extracellular vestibule, then diffusing into the orthosteric pocket. In contrast, fentanyl may take a novel route; first partitioning into the membrane, before accessing the orthosteric site by diffusing through a ligand-induced gap between the transmembrane helices. In electrophysiological recordings fentanyl-induced currents returned after washout, suggesting fentanyl deposits in the lipid membrane. However, mutation of residues forming the potential MOPr transmembrane access site did not alter fentanyl’s pharmacological profile *in vitro*. A high local concentration of fentanyl in the lipid membrane, possibly in combination with a novel lipophilic binding route, may explain the high potency and lower susceptibility of fentanyl to reversal by naloxone.

## Introduction

The synthetic opioid agonist, fentanyl, is used medicinally as a powerful, fast-acting analgesic. However, fentanyl and analogues (fentanyls) have increasingly appeared in the illicit drug market (1, 2); this has been associated with a dramatic rise in acute opioid overdose deaths involving fentanyls (3). Concerningly, there are increasing reports that fentanyl overdose requires higher doses of the antagonist naloxone to reverse, compared to heroin (4-9). Indeed, we have recently shown that naloxone reverses fentanyl-induced respiratory depression in mice less readily than that induced by morphine (10). This finding is at odds with classical receptor theory, as under competitive conditions the degree of antagonism depends only on the affinity and concentration of the antagonist, not the potency of the agonist (11). Fentanyls, therefore, are a major public health concern, and exhibit a unique pharmacology which is incompletely understood.

*In vitro,* there is a discrepancy between the relative potencies of fentanyl and morphine in experiments performed in membrane homogenate and intact cell systems (12). In membrane homogenates, fentanyl and morphine exhibit similar affinity of binding to the µ-opioid receptor (MOPr), both in the absence and presence of Na^+^ ions (13-15), whilst in membrane homogenate studies of receptor activation using GTPγS binding the potency of fentanyl has been reported to be less than 2 fold greater than that of morphine (14-16). In marked contrast, in intact cells fentanyl is some 5–50 fold more potent than morphine (16-20). This difference is further exacerbated *in vivo* (12); fentanyl has been reported as over 100 fold more potent than morphine in producing anti-nociception in mouse (21) and rat (22, 23), and 50 fold more potent in producing analgesia in humans, compared to morphine (24). Fentanyl also exhibits a fast onset of action compared to other opioid agonists; a property attributed to its high lipophilicity allowing rapid penetration across the blood-brain-barrier.

MOPr, the GPCR which mediates the pharmacological effects of fentanyl (10), has a deep aqueous binding pocket for orthosteric ligands which is shielded from the extracellular milieu by three extracellular loops (ECLs) and from the lipid bilayer by the seven transmembrane helices (TMDs) (Supplementary Figure S2). It is generally assumed that GPCR ligands bind to the orthosteric site directly from the extracellular aqueous phase (25-27). However, some highly lipophilic ligands are able to access the orthosteric pocket by diffusing through the membrane and the TMDs (28-31).

Therefore, we propose that fentanyl’s differing potencies dependent on the membrane environment may be explained by the unusual chemical properties of fentanyl. Firstly, fentanyls are highly lipophilic compared to other opioids (32, 33) therefore, in intact cells, may partition into the bilayer, increasing the drug concentration around the receptor (34-36). Secondly, fentanyls have an elongated structure with a central protonatable nitrogen and 6 rotatable bonds, compared to the rigid ring structure of morphinan compounds (Supplementary Figure S1). This flexible structure may facilitate a novel binding process, distinct from that of morphinans, whereby fentanyl binds to the MOPr *via* the lipid bilayer.

Long timescale all-atom molecular dynamics (MD) simulations have been used to capture small molecules binding to GPCRs (26, 27). However, capturing a rare event such as ligand binding usually requires millisecond timescale simulations using specially-designed machines (37). Coarse grained (CG) MD can be utilised to overcome these sampling issues (38-40). In CG MD, rather than representing each individual atom as a defined bead, groups of atoms are represented as a single bead describing the overall properties of the chemical group. This lower resolution representation allows the conformational landscape to be efficiently sampled and the capture of rare events such as ligand binding (41, 42).

To determine how fentanyls and morphinans might access and bind to MOPr, we first employed unbiased CG MD simulations to predict how different opioids bind and unbind. We quantified our observations using potential of mean force (PMF) calculations. Following identification of possible binding routes *in silico*, we then explored fentanyl’s ability to partition into the membrane and interact with endogenous MOPrs in locus coeruleus (LC) neurons and with mutated MOPrs expressed in AtT20 cells.

## Materials and Methods

### *In Silico* Studies

#### System Set-Up

The MOPr model was taken from the inactive, antagonist-bound crystal structure (43) (PDB: 4DKL), with the T4 lysozyme and ligands removed, and the missing intracellular loop 3 modelled using Insight II, as described in (44). The protein structure coordinates were then converted to coarse-grained MARTINI 2.2 representation using the *martinize* script (45). In order to maintain the overall structure of the protein, the secondary structure was constrained using an elastic network between backbone (BB) beads (Supplementary Figure S6); elastic bonds with a force constant of 100 kJ mol^−1^nm^−2^ were defined between BB_i_-BB_i+4_ helix atoms, BB_i_-BB_i+10_ helix atoms, and BB atom pairs with low root mean square fluctuation and highly correlated motion as determined from all-atom MD simulations (44, 46). Flexibility of loop regions is crucial for drug binding and GPCR activation (47), therefore no elastic network was applied to the loops. Root mean square deviation (RMSD) of the protein backbone, along with the distances between the extracellular ends of each TMD, were measured during initial 1 µs simulations and compared to that obtained in all-atom MD simulations, to determine that the secondary structure of the protein was maintained (Supplementary Figures S6, S7). We further compared our elastic network with the automated elastic networks generated by *martinize* (45) and ElNeDyn (48), or our MOPr model with no elastic network applied (Supplementary Figure S6). We judged that our elastic network conferred similar dynamics to martini and ElNeDyn, without the disadvantage of adding rigidity to physiologically flexible loops. All MD simulations were run using GROMACS 2019.2 (49).

To parameterise morphine and fentanyl in MARTINI, firstly, 1 µs all-atom MD simulations of fentanyl or morphine in water and 0.15 M NaCl were conducted under the Amber ff99SB-ildn force field (50). Ligands were parameterised using acpype/Antechamber and the General Amber Force Field (51). Atom-to-bead mapping for morphine and fentanyl was then created as shown in Supplementary Figure S1, whereby each atom was assigned to an appropriate coarse-grain bead. The CG ligands were then solvated in water and 0.15 M NaCl, energy minimized for 10,000 steps using the steepest descents algorithm, box dimensions and temperature equilibrated, and then production MD was run for 1 µs Bond lengths and angles were measured and compared to the all-atom simulations, to determine appropriate mapping and bonded terms (Supplementary Figures S8, S9).

#### Unbiased CG Simulations

The CG MOPr model was then embedded in a POPC:POPE:cholesterol lipid bilayer [ratio 5:5:1, comparable to that found in mammalian cells (52, 53)] using the *insane* script (54), and solvated in water, 0.15 M NaCl and 6 molecules of opioid ligand. The starting size of the system box was 15 × 15 × 15 nm^3^. Systems were first energy minimized over 50,000 steps using the steepest descents algorithm, then equilibrated under NVT ensemble and then NPT ensembles, before production MD simulations were run at 310 K with a 10 fs timestep. The temperature and pressure were controlled by the V-rescale thermostat and Parrinello-Rahman barostat, respectively. Simulations were performed for up to 5 µs; the exact simulation lengths for each ligand are shown in Supplementary Table S1.

All simulations were analysed using the GROMACS suite of tools (49). Unless otherwise stated, all analyses were performed using the entire production trajectories. Data were plotted in GraphPad Prism v8, and images made in VMD (55).

#### Free Energy Calculations

The overall process for determining the free energy of binding (ΔG_binding_) by steered MD and umbrella sampling is depicted in Supplementary Figure S10. Steered MD utilizes a pulling force to generate a simulation of the ligand unbinding from the membrane or MOPr. Overlapping snapshots along this unbinding simulation then serve as the starting points for umbrella sampling simulations. During umbrella sampling several independent simulations are performed, one for each snapshot along the unbinding pathway. The ligand is restrained within its starting snapshot, allowing the ligand to fully and efficiently explore the conformational space in this defined region. From these independent simulations of the overlapping snapshots the free energy of binding across the entire unbinding pathway can then be extracted.

For the membrane/solvent partitioning calculations, systems were set up with small (5 × 5 × 10 nm^3^) membrane patches containing 32 POPE, 32 POPC and 6 cholesterol molecules (ratio 5:5:1), and solvated in 0.15 M NaCl. One molecule of either protonated fentanyl, neutral fentanyl, protonated morphine or neutral morphine was placed in the bilayer center. The systems were minimized for 50,000 steps, keeping the ligand restrained. To generate the starting conformations for umbrella sampling, steered MD simulations were performed. Ligands were pulled from the bilayer center into the solvent (56), in a direction defined by the vector between the centers of mass of the ligand and the PO4 lipid beads, at a rate of 0.1 nm ns^−1^ and a force constant of 1,000 kJ mol^−1^ nm^−2^.

For the ligand binding calculations, the final frames from the unbiased CG simulations with morphine or fentanyl bound in the orthosteric pocket were taken as the starting conformations. All other unbound ligands were removed, and the receptor-ligand complex was re-embedded in a smaller lipid bilayer (10 × 10 × 10 nm^3^). Steered MD simulations were performed to generate the starting conformations for umbrella sampling. In each case, separate simulations were performed to pull morphine or fentanyl from the orthosteric pocket along 1) the aqueous/extracellular route, and 2) the lipophilic/transmembrane domain route. The reaction coordinate was defined as the distance between the center of mass of the ligand and the receptor. Ligands were pulled at a rate of 0.1 nm ns^−1^ and a force constant of 1,000 kJ mol^−1^nm^−2^, with a 1,000 kJ mol^−1^ nm^−2^ position restraint on 4 backbone beads (D114^2.50^, D147^3.32^, N150^3.35^ and S154^3.39^) of the MOPr to prevent translation or rotation of the receptor. These restraints should have no discernable impact on the reported binding energies.

The starting conformations for umbrella sampling were extracted from these steered MD trajectories at 0.05 nm intervals along the reaction coordinate, generating ∼80 umbrella sampling windows for each calculation. Each was subjected to 1 µs MD simulations, with a harmonic restraint of 1,000 kJ mol^−1^ nm^−2^ to maintain the separation between the centers of mass of the ligand and PO4 beads (membrane partitioning calculations) or protein (ligand binding calculations). The PMFs were then extracted using the Weighted Histogram Analysis Method (WHAM) in GROMACS (57), which inherently accounts for the imposed restraints. PMFs were plotted as the average profile with statistical error calculated from bootstrap analysis. For the ligand binding calculations, ΔG_binding_ for each ligand in each binding pathway was calculated as the difference between the ligand-bound and final unbound states.

### Experimental Studies

#### Brain Slice Preparation

Male Wistar rats (4 weeks old) were anaesthetized through i.p. injection of 160 mg kg^−1^ ketamine and 20 mg kg^−1^ xylazine and then decapitated. Brains were then removed and submerged in an ice-cold cutting solution containing (in mM): 20 NaCl, 2.5 KCl, 1.6 NaH_2_PO_4_, 7 MgCl_2_, 85 sucrose, 25 D-glucose, 60 NaHCO_3_ and 0.5 CaCl_2_, saturated with 95% O_2_/5% CO_2_. Horizontal 230 µm thick brain slices containing the locus coeruleus (LC) were then prepared using a vibratome. Slices were subsequently incubated in a warm (32°C) artificial cerebrospinal fluid (aCSF) containing (in mM): 125 NaCl, 2.5 KCl, 1.2 NaH_2_PO_4_, 1.2 MgCl_2_, 11.1 D-glucose, 21.4 NaHCO_3_, 2.4 CaCl_2_ and 0.1 ascorbic acid, saturated with 95% O_2_/5% CO_2_ and were left to equilibrate for at least 1 h.

All animal care and experimental procedures were in accordance with the UK Animals (Scientific Procedures) Act 1986, the European Communities Council Directive (2010/63/EU), the ARRIVE guidelines (58) and the University of Bath ethical review document.

#### Whole-Cell Patch-Clamp Electrophysiological Recordings

Rat brain slices were transferred to a recording chamber and superfused with continuous flow (2.5 ml min^−1^) of warm (32°C) aCSF. Whole-cell recordings were made using recording electrodes (3–5 MΩ) containing an internal solution of (in mM): 115 potassium gluconate, 10 HEPES, 11 EGTA, 2 MgCl_2_, 10 NaCl, 2 MgATP, and 0.25 Na_2_GTP, and pH 7.3 and with an osmolarity of 270 mOsm.L^−1^. LC neurones were voltage-clamped at −60 mV, with a correction made for a −12 mV junction potential.

All drugs were applied in the superfusing solution at known concentrations. Fentanyl and morphine were applied at concentrations determined to evoke equivalent submaximal responses (EC_80_) in rat LC neurones (100 nM and 1 µM respectively, data not shown). Opioids were applied for 10 min to allow for evoked outward GIRK currents to rise to a steady state. Subsequently, naloxone (30 nM) was applied in superfusing solution in combination with fentanyl or morphine for 15 min. At this concentration, naloxone was demonstrated to partially reverse GIRK currents evoked by morphine (1 µM) and fentanyl (100 nM) in LC neurones to similar levels. Drug-free aCSF was then superfused over the slice and the GIRK current was tracked for 10 min, before 10 µM naloxone was applied to fully reverse opioid-induced GIRK currents.

The data were tested for normality by the Shapiro-Wilk test (passed, W = 0.9583, *p* = 0.7962) and visual examination of the QQ plot. Therefore we used the parametric paired two-tailed *t*-test to determine statistical differences between conditions. Values are presented as mean ± SEM where N = 5. Each experimental replicate (N) was run in brain slices derived from separate animals.

#### MOPr Transfection and Cell Culture

Wild type AtT20 cells stably expressing human MOPr were a gift from Marina Santiago (Macquarie University, Australia). An AtT20 stable cell line expressing a MOPr double mutant, MOPr^P309R−E310R^ was generated using the Invitrogen Flp-In protocol. Hygromycin-resistant and zeocin-sensitive clones were selected and expanded.

Cells were cultured in DMEM supplemented with 10% FBS, 50 U/mL penicillin, 0.5 mg/ml streptomycin (P/S) and 80 μg/ml hygromycin B for the maintenance of transfected cells. Incubator conditions were maintained at 5% CO2, 37°C and high relative humidity.

#### Membrane Potential Assay

The protocol followed was as previously described (59). AtT20 cells at ∼90% confluency were detached using trypsin/EDTA and resuspended in Leibovitz’s L-15 media supplemented with P/S 1%, FBS 1% and 15 mM glucose. In poly-L-lysine coated black 96-well clear flat-bottom plates, 90 µL of the cell suspension were seeded in each well and incubated overnight in an air-only incubator. One hour prior to the experiment, 90 µL of the fluorescent blue membrane potential dye was loaded into each well. Blue dye as well as all drug dilutions were prepared in a low potassium buffer. Fentanyl hydrochloride was purchased from Tocris, morphine hydrochloride from Macfarlan Smith, and naloxone hydrochloride was from Sigma-Aldrich.

Fluorescence was measured using the FlexStation 3 Multi-Mode Microplate Reader (Molecular Devices) where cells were excited at a wavelength of 530 nm, emission measured at 565 nm and readings were taken every 2 s and continued until agonist or antagonist responses had reached a steady state. The amplitude of responses was calculated as the percentage change from baseline fluorescence readings. Baseline readings were taken for 30 s before 10 µL of agonist or buffer was injected. The response was measured at the lowest reduction in signal. Responses from wells that received buffer only were subtracted. The change in the signal produced by the addition of buffer alone was less than 5% of the baseline. Background fluorescence in wells with cells only or dye only was very low and regarded as negligible. For the antagonist reversal experiments, baseline readings were taken for 30 s prior to the addition of 10 µL of the submaximal concentration of each agonist (morphine 1 µM and fentanyl 20 nM). These agonist concentrations were chosen to produce comparable amplitudes of response for morphine and fentanyl in wildtype MOPr cells (see Figure 7C and Figure 7E). When agonist response reached steady state (60 s post agonist addition), 10 µL of naloxone (final concentration 10 µM) was used to reverse the signal. Assays were conducted in duplicate and mean data from 5 separate experiments are presented. The concentration-response data were analysed by non-linear regression (GraphPad Prism v8).

## Results

### Fentanyl Partitions Into the Lipid Membrane

We built molecular systems of the MOPr (43, 44, 46) (PDB: 4DKL) in a solvated membrane using the coarse grained MARTINI 2.2 force field, added 6 molecules of either protonated fentanyl, neutral fentanyl, protonated morphine or neutral morphine (Supplementary Figure S1) to the solvent and ran 3–6 independent repeats of 1–5 µs unbiased CG MD simulations to allow the ligands to bind to the MOPr (Supplementary Table S1).

**FIGURE 1:**
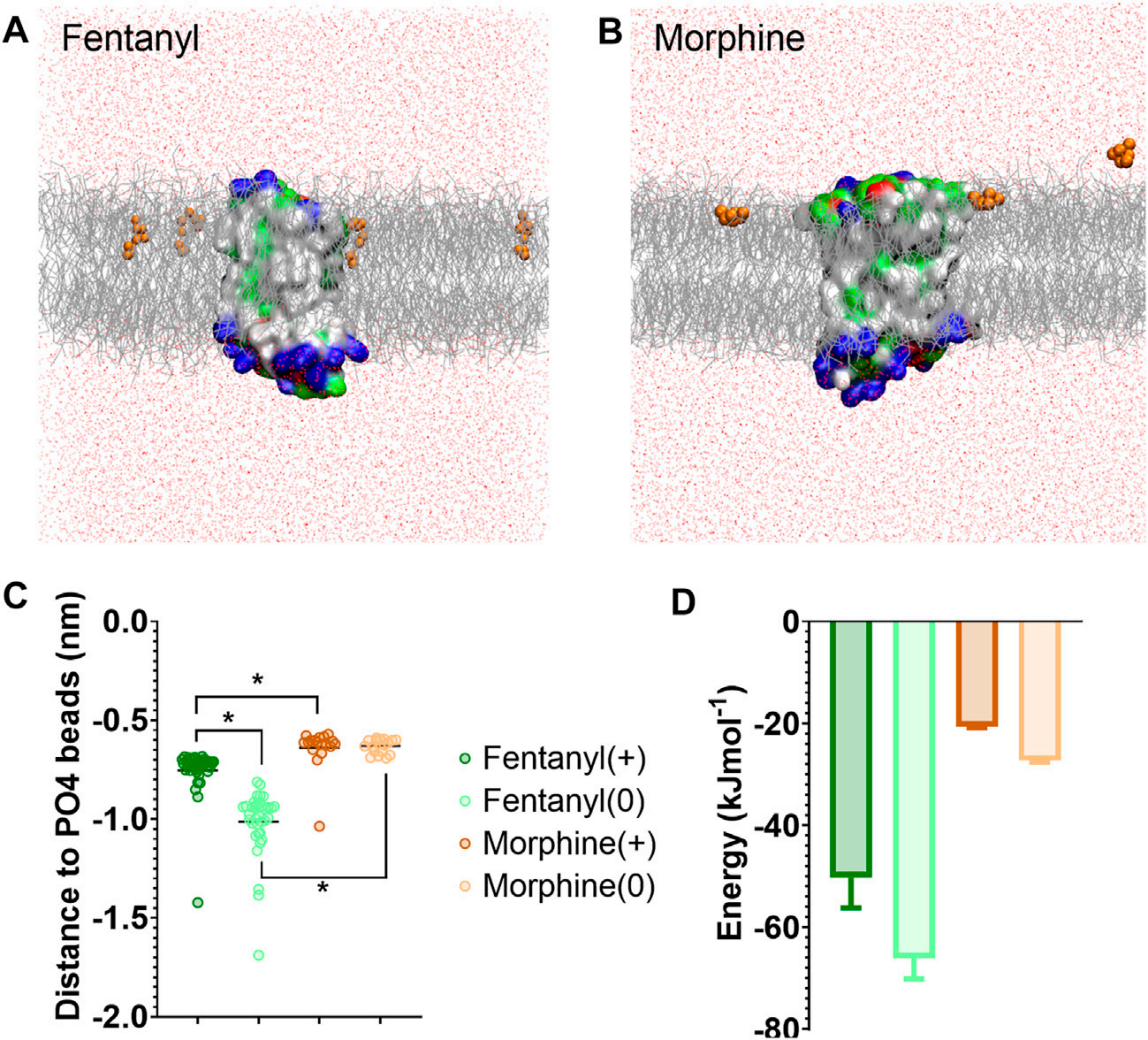
Differences in how opioid ligands partition into the lipid bilayer. **(A)** Fentanyl molecules (orange) rapidly partitioned into the lipid membrane (grey). **(B)** Morphine molecules (orange) did not fully enter the lipid membrane (grey) but interacted with the charged lipid headgroups. Note while ligands can appear on either side of the bilayer due to the periodic boundary conditions applied in these simulations, for clarity only ligands in the upper leaflet of the membrane are shown. In no simulation did a ligand travel all the way through the bilayer. The protein is coloured according to residue properties (hydrophobic; grey, polar; green, acidic; red, basic; blue). **(C)** Distance between the center of mass of the ligand and the phosphate head groups (PO4 beads) of the lipid bilayer. Both the charged and neutral forms of fentanyl partitioned significantly deeper in the membrane than morphine. *p < 0.05, one-way ANOVA. Each data point represents the average distance between a fentanyl molecule and the PO4 beads over the entire simulation. **(D)** Free energy change for ligands moving between the bilayer center and the aqueous solvent. Calculated from PMF profiles shown in Supplementary Figure S3. Data plotted as mean ± error calculated from bootstrap analysis.

We first characterised how the protonated and neutral forms of fentanyl and morphine interacted with the membrane. In all simulations, fentanyl and morphine rapidly diffused from the solvent to interact with the bilayer. Both the protonated and neutral fentanyl molecules fully partitioned into the membrane (Figure 1A), with the neutral form of the ligand penetrating deeper into the bilayer centre (Figure 1C and Supplementary Figure S3). In contrast morphine interacted only with the phosphate head groups at the lipid-solvent interface (Figure 1B), and neither the protonated nor neutral form of the ligand partitioned into the bilayer (Figure 1C).

To further quantify the propensity for fentanyl and morphine to partition between the aqueous and lipid phase, we performed steered MD and umbrella sampling to calculate the free energy change (ΔG) of membrane partitioning. Steered MD uses an external force to “pull” the ligand away from the center of the membrane (56), creating a trajectory of the ligand moving between the lipid and aqueous solvent from which umbrella sampling can be performed to extract PMF profiles. Using these PMFs, ΔG can be calculated as the free energy difference between the ligand residing in the bilayer center verses the aqueous solvent. The resulting ΔG values are shown in Figure 1D, and the PMF profiles in Supplementary Figure S3.

The calculated ΔG for membrane partitioning for the protonated and neutral forms of fentanyl were −50.3 ± 6.0 kJmol^−1^ and −66.1 ± 4.1 kJmol^−1^, respectively. Whereas, the values for morphine showed a much smaller free energy difference (protonated; −20.6 ± 0.3 kJmol^−1^, neutral; −27.3 ± 0.3 kJmol^−1^). The spontaneous membrane partitioning exhibited by fentanyl in the unbiased CG simulations, along with this greater free energy change in partitioning between the lipid and the aqueous solvent, supported our unbiased simulations which showed that fentanyl has a greater propensity to concentrate in the cell membrane than morphine.

**FIGURE 2:**
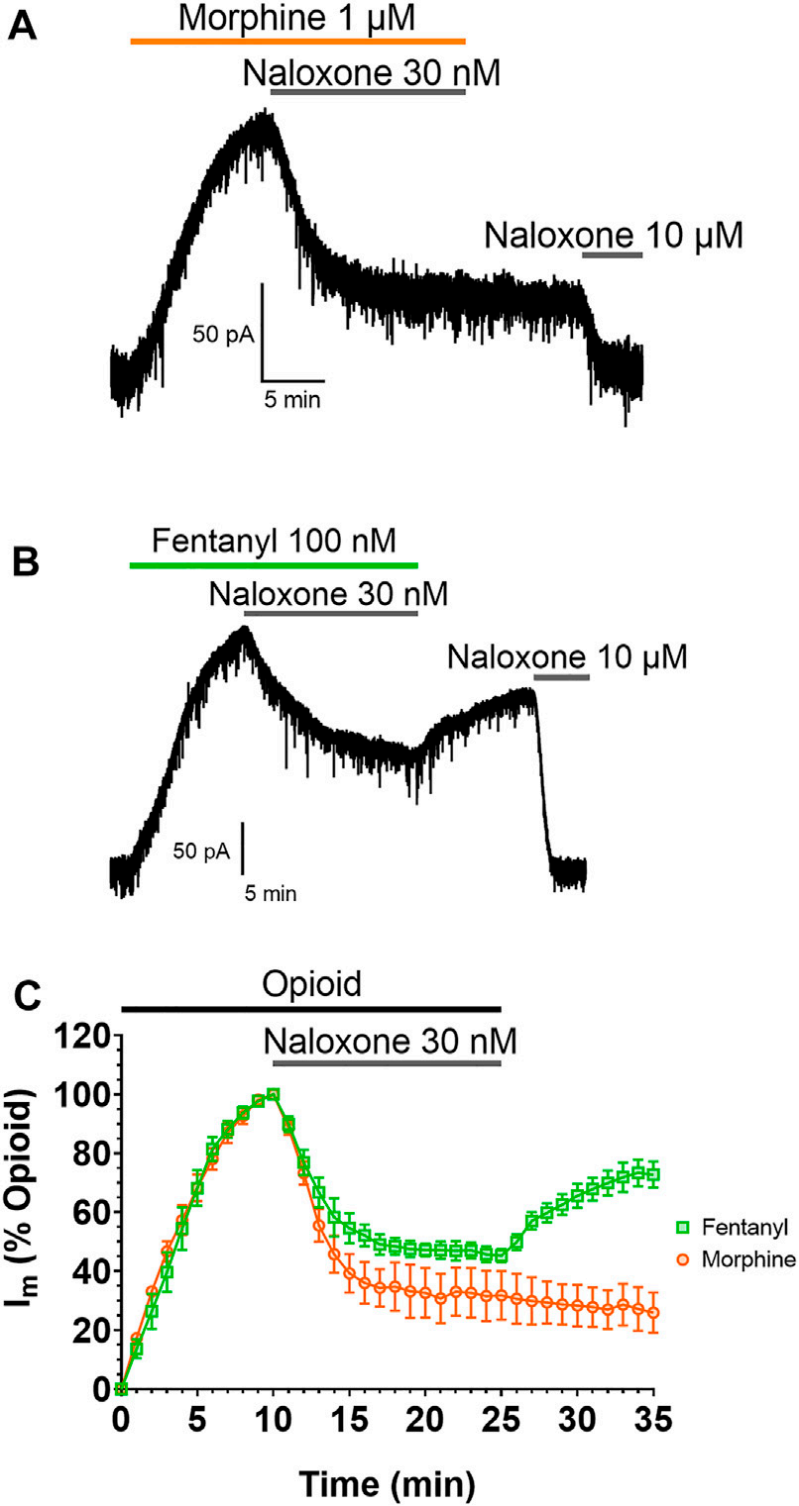
Fentanyl, but not morphine, reasserts its action after washout Representative recordings showing GIRK currents evoked by submaximal concentrations of **(A)** morphine and **(B)** fentanyl in rat locus coeruleus (LC) neurones. Opioid-evoked currents were partially reversed by the coaddition of 30 nM naloxone, before drug-free aCSF was applied to the cells for 10 min. 10 µM naloxone was then applied to reverse remaining opioid-evoked currents. **(C)**. Combined data from experiments presented in **(A,B)**. Opioid-evoked membrane currents (I_m_) are expressed relative to the peak current evoked by each agonist in each cell, mean ± SEM, N = 5.

The impact of this membrane partitioning on the pharmacological characteristics of fentanyl was explored using brain slice electrophysiology. Whole-cell electrophysiological recordings of opioid-evoked G protein activated inwardly rectifying potassium (GIRK) currents were made from rat LC neurons voltage-clamped at −60 mV (60). Slices were treated with submaximal concentrations (EC_80_) of morphine (1 µM) or fentanyl (100 nM) for 10 min, before the coapplication of 30 nM naloxone for 15 min to partially reverse the responses of the agonists (Figure 2). Slices were then superfused with drug-free aCSF for 10 min to remove the agonists and antagonists from the extracellular space before the remaining opioid-evoked current was fully reversed by application of 10 µM naloxone. Figures 2A,B show representative traces for the morphine and fentanyl-induced currents. Coaddition of 30 nM naloxone partially reversed both morphine- and fentanyl-evoked GIRK currents to a similar degree (Figure 2C). After partial reversal by 30 nM naloxone and subsequent wash-out of both morphine and naloxone the morphine-evoked currents steadily declined (Figure 2C). In stark contrast, we observed a clear reassertion of fentanyl-evoked currents upon wash-out of fentanyl and naloxone (Figure 2C). The magnitude of the fentanyl-evoked current (expressed as % peak fentanyl response) significantly increased from 45 ± 2% after 15 min application of 30 nM naloxone, to 72 ± 4% after 10 min wash-out (*p* = 0.0006). This observation, combined with the simulation data above, suggested that fentanyl was not fully washed out of the tissue due to it partitioning into the lipid membrane.

We excluded the possibility of the response on wash-out being due to fentanyl having adhered to the tubing and then leaching into the drug-free perfusate during washout (see Supplementary Information).

### Fentanyl can Bind to MOPr *via* the Lipid Phase and the Transmembrane Helices

For the remaining analyses, we focused on the simulations of the protonated ligands, as the charged species is required to form the canonical amine—D147^3.32^ salt bridge essential for opioid ligand binding within the orthosteric pocket (61) (Supplementary Figure S2).

**FIGURE 3:**
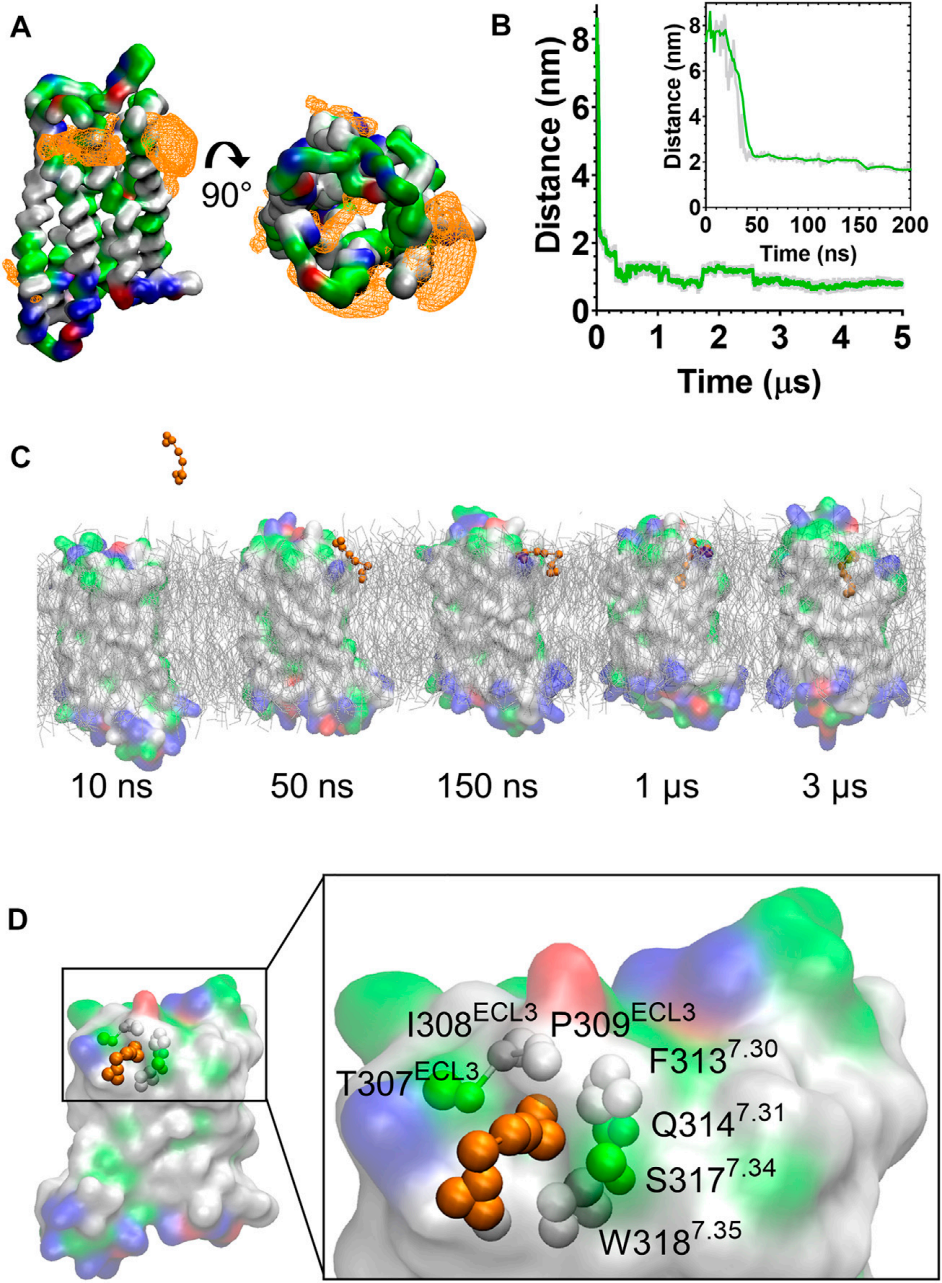
Fentanyl binds to the MOPr from the lipid phase, *via* a gap between TM6 and TM7. **(A)** Ligand density maps averaged over the 5 µs simulation, show fentanyl densities around the receptor transmembrane domains and within the orthosteric pocket (orange). The protein is coloured according to residue properties (hydrophobic; grey, polar; green, acidic; red, basic; blue). **(B)** Distance between the Qd bead of fentanyl and the SC1 bead of D147^3.32^ over the entire 5 µs and in the first 200 ns (inset). Data are presented as the raw values (grey) and moving average over 10 frames (green). **(C)** Snapshots from the unbiased simulation of fentanyl binding to MOPr. Fentanyl moved from the aqueous solvent into the lipid bilayer, then interacted with the MOPr transmembrane domains and induced the formation of a gap between TM6 and 7, through which fentanyl accessed the orthosteric site. **(D)** Fentanyl at the TM6/7 interface. Fentanyl is depicted as orange beads, and the residues comprising the lipid entry gap as coloured beads.

In the CG MD simulations fentanyl molecules in the lipid bilayer appeared to congregate around MOPr. We therefore constructed ligand density maps across all the fentanyl simulations (Figure 3A), using the VMD VolMap tool (55). Fentanyl molecules clustered around the receptor helices in the upper leaflet of the membrane, with densities determined on the lipid-facing sides of the TM1/2, TM6/7 and TM7/1 interfaces.

Most notably, we also observed fentanyl diffusing through MOPr to the orthosteric binding pocket *via* a novel lipophilic pathway (see Supplementary Movie S1). Snapshots from the MD simulation (Figure 3C and Supplementary Figure S4) showed fentanyl first partitioning into the lipid bilayer, then interacting with a ligand-induced gap at the TM6/7 interface, and finally accessing the orthosteric site by diffusing through this gap in the MOPr helices. The fentanyl molecule took 3 µs to diffuse across the receptor TM domains to the orthosteric site (Figure 3B).

The TM6/7 interface and the gap induced by the fentanyl molecule is shown in Figure 3D. This interface comprises hydrophobic and polar residues from TM6 and 7, as well as ECL3. Specifically, the relatively small side chains of L305^6.60^, T307^ECL3^, I308^ECL3^ and P309^ECL3^ allowed formation of a pore through which the phenethyl group of fentanyl (represented by the F1, F2 and F3 beads, see Supplementary Figure S1) was observed to access the receptor orthosteric pocket. Meanwhile, the aromatic side chain of W318^7.35^ stabilised the position of fentanyl’s N-phenyl-propanamide (represented by the F7, F8 and F9 beads, see Supplementary Figure S1).

### Morphine Binds to MOPr *via* the Aqueous Phase and an Extracellular Vestibule Site

**FIGURE 4:**
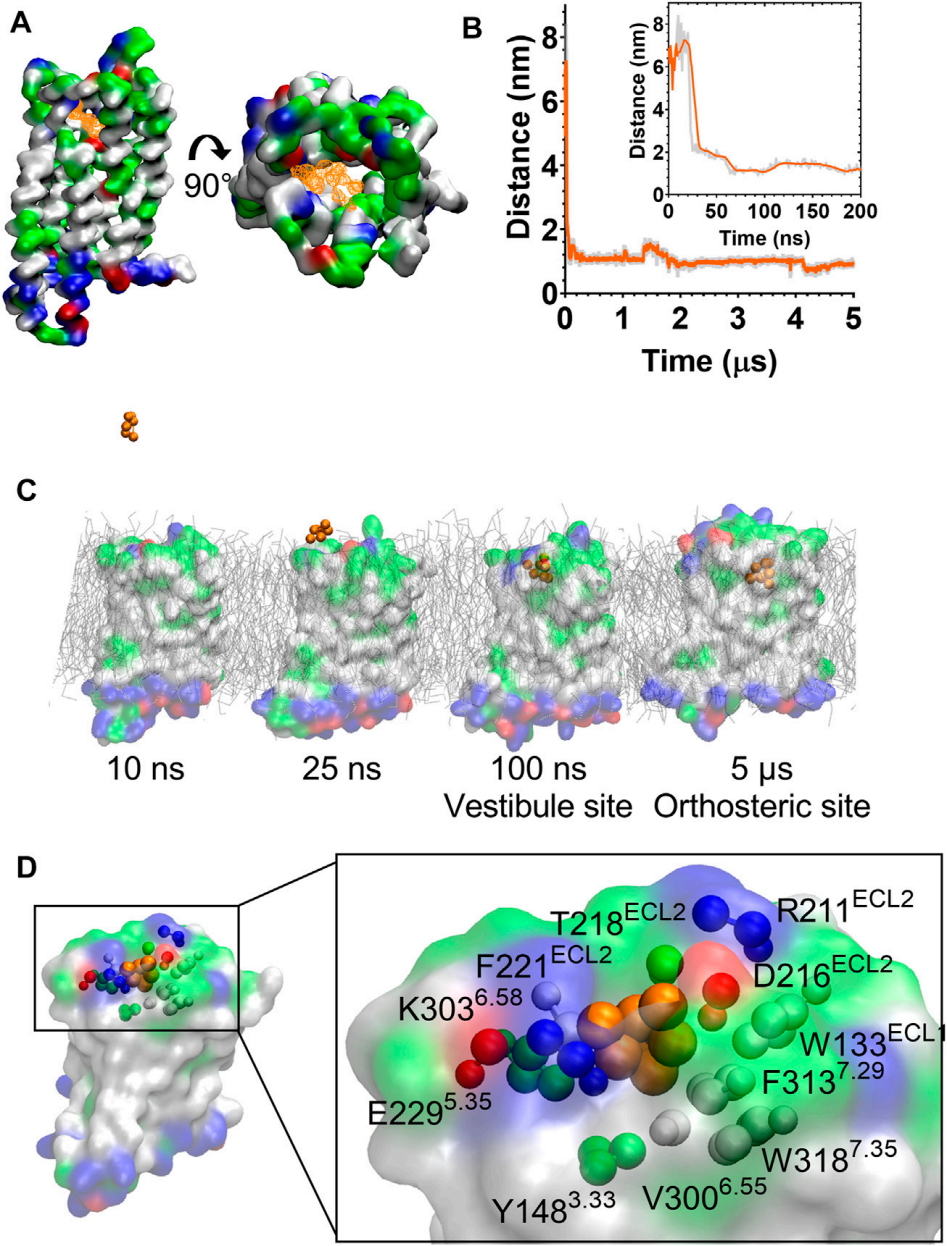
Morphine binds to the MOPr from the aqueous phase, *via* an extracellular vestibule site. **(A)** Ligand density maps averaged over the 5 µs simulation, show morphine densities above and within the orthosteric pocket (orange). The protein is coloured according to residue properties (hydrophobic; grey, polar; green, acidic; red, basic; blue). **(B)** Distance between the Qd bead of morphine and the SC1 bead of D147^3.32^ over the entire 5 µs and in the first 200 ns (inset). Data are presented as the raw values (grey) and moving average over 10 frames (orange). **(C)** Snapshots from the unbiased simulation of morphine binding to MOPr. Morphine moved from the aqueous solvent to an extracellular vestibule and finally the orthosteric site. **(D)** Morphine in the extracellular vestibule site. Morphine is depicted as orange beads, and the residues comprising the vestibule site as coloured beads.

During the unbiased CG simulations, we observed morphine spontaneously binding to the MOPr *via* the canonical aqueous pathway (see Supplementary Movie S2). Ligand density maps showed a density for a morphine molecule in the extracellular portion of the MOPr; above and within the orthosteric binding site (Figure 4A). Plotting the distance between the charged Qd bead of morphine and the side chain bead of D147^3.32^ showed that the ligand rapidly diffuses from the aqueous solvent to interact with the extracellular surface of MOPr within the first 50 ns of the CG simulation (Figure 4B). Morphine maintained stable interactions with this extracellular site for 4.2 µs, before finally moving deeper into the orthosteric binding pocket. Figure 4C and Supplementary Figure S4 show snapshots of morphine travelling along this canonical aqueous binding pathway, with it initially binding to the extracellular vestibule site and then finally binding within the orthosteric pocket.

The extracellular vestibule site is shown in Figure 4D, comprising primarily polar or charged residue side chains in ECL2 and the extracellular ends of TMs 5, 6 and 7. This extracellular vestibule site appears to be a conserved feature of small molecule binding to Class A GPCRs, having previously been highlighted in MD simulations of the β1 and β2 adrenoceptors (26), M3 muscarinic receptor (27), adenosine A_2A_ receptor (41) and oliceridine binding to the MOPr (25).

### Calculation of the Relative Binding Energies in the Aqueous and Lipophilic Access Routes

Next, we sought to further characterize the aqueous and lipid access pathways by calculation of the free energy of binding (ΔG_binding_) for each ligand in each pathway.

**FIGURE 5:**
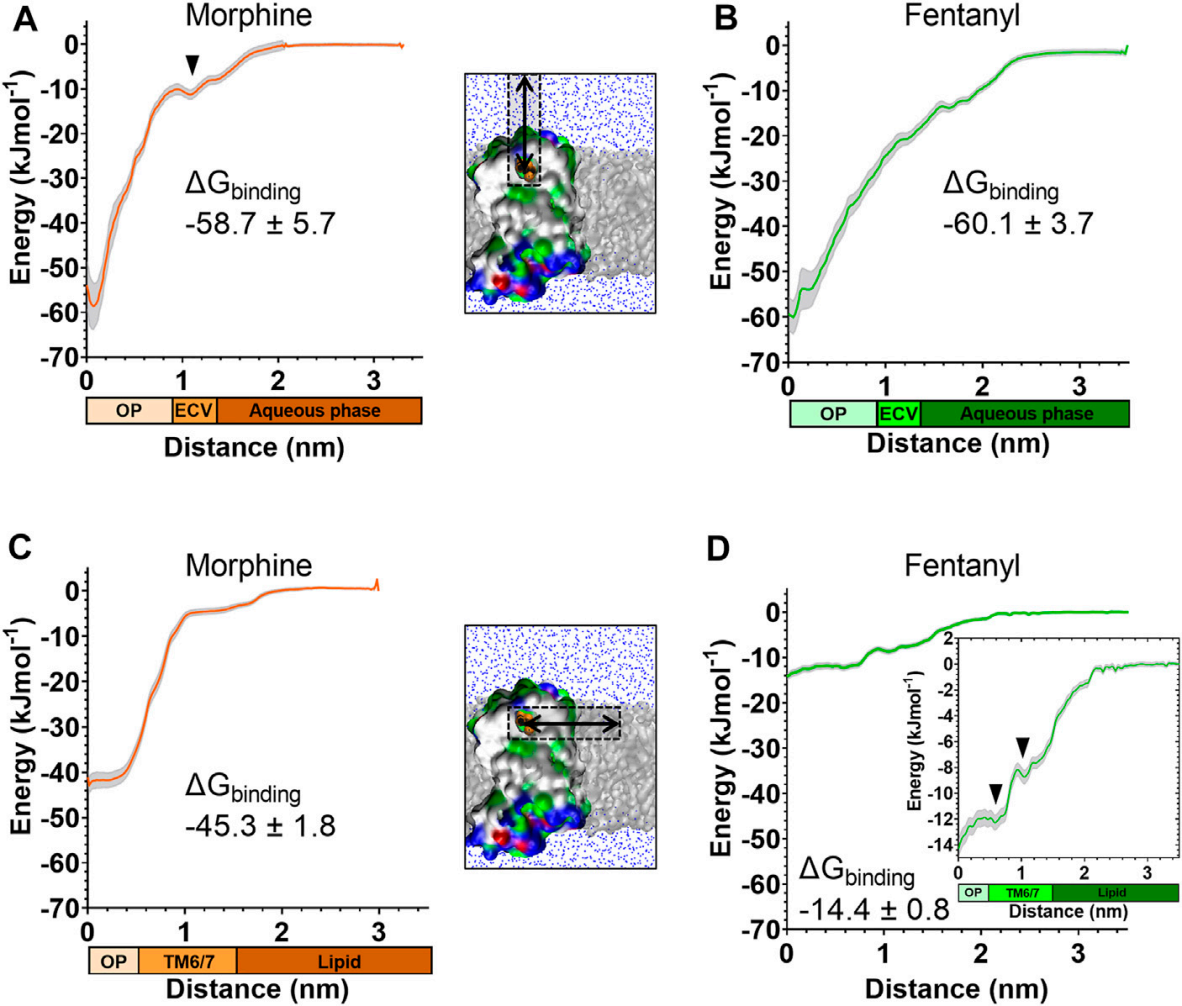
Free energy calculations for ligand binding pathways Steered MD was used to recreate the spontaneous binding events reported in Figures 3, 4. Umbrella sampling and the weighted histogram analysis method were then employed to determine the free energy of binding for each ligand in each pathway. In all plots the distance along the reaction coordinate is defined as the distance between the centre of mass of the ligand and receptor. Coloured bars beneath the x-axes indicate the orthosteric pocket (OP), extracellular vestibule (ECV), TM6/7 interface, lipid and aqueous phases. Data are plotted as an average (coloured line) and statistical error (grey), calculated from bootstrap analysis. ΔG_binding_ is expressed as mean ± statistical error. **(A)** PMF profile for morphine binding *via* the aqueous pathway. **(B)** PMF profile for fentanyl binding *via* the aqueous pathway. **(C)** PMF profile for morphine binding *via* the lipid pathway. **(D)** PMF profile for fentanyl binding *via* the lipid pathway. Inset shows the same data with expanded y axis.

Starting from the final frames of the simulations where fentanyl (Figure 3C) or morphine (Figure 4C) bound in the orthosteric site, steered MD simulations were performed to recreate the aqueous and lipid binding routes for each ligand. Ligands were “pulled” from the orthosteric site along either the aqueous or lipid access route, generating a trajectory from which starting conformations for umbrella sampling could be generated. The resulting PMF profiles are presented in Figure 5, along with the calculated ΔG_binding_ values for each ligand in each binding pathway. Histograms are shown in Supplementary Figure S5. Here, ΔG_binding_ represents the free energy difference between the ligand-bound MOPr and the unbound ligand residing in either the aqueous solvent (Figures 5A,B) or the lipid membrane (Figures 5C,D).

The PMF profiles for morphine and fentanyl binding *via* the aqueous pathway are shown in Figures 5A,B, respectively. The calculated ΔG_binding_ for each ligand was similar (-58.7 ± 5.7 kJmol^-1^ for morphine, −60.1 ± 3.7 kJmol^-1^ for fentanyl), suggesting that both ligands can bind *via* this aqueous route with similar ease. In the profile for morphine binding a small local minimum can be seen between 1.0–1.3 nm, indicating the extracellular vestibule site identified in the unbiased MD simulations (Figure 4D). In the profile for fentanyl binding no small local minimum indicative of binding to the extracellular vestibule was apparent.

The PMF profiles for morphine and fentanyl binding *via* the lipid access pathway are shown in Figures 5C,D. For morphine, the PMF profile followed a steep curve, with a calculated ΔG_binding_ of −45.3 ± 1.8 kJmol−^1^. In contrast, the fentanyl ΔG_binding_ was significantly lower (−14.4 ± 0.8 kJmol^−1^), with two local minima at 0–0.8 nm and 1.1–1.5 nm, corresponding to the orthosteric site and the TM6/7 interface (Figure 3D) on the lipid-facing side of the helices, respectively.

### Comparison of Free Energy Landscapes for Morphine and Fentanyl

**FIGURE 6:**
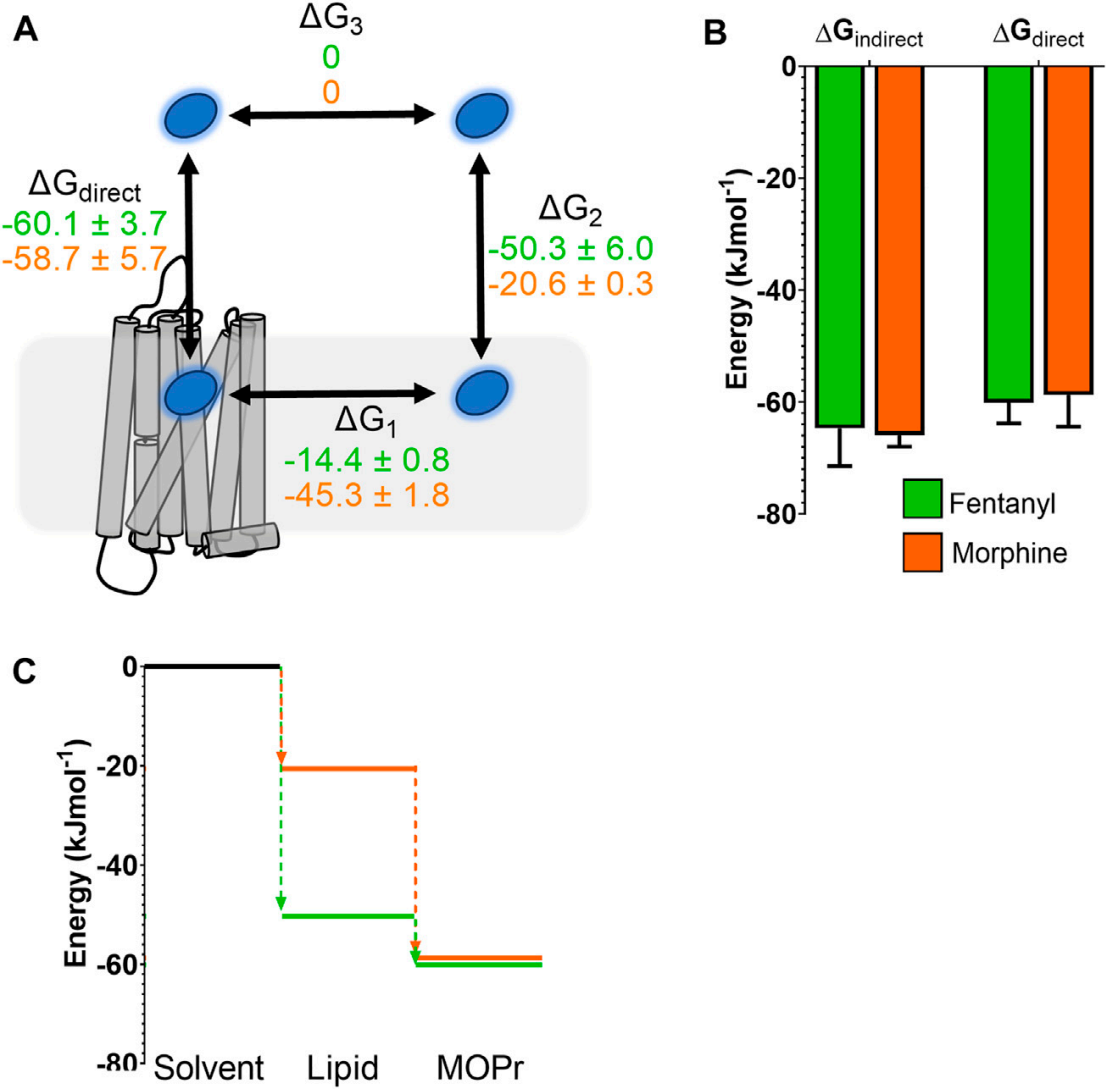
Comparison of free energy landscapes for fentanyl and morphine binding to the MOPr. **(A)** Thermodynamic cycle for opioid ligand binding to MOPr; either by the direct, aqueous pathway (ΔG_direct_) or *via* the lipid membrane (ΔG_1_ + ΔG_2_). Values for protonated fentanyl (green) and protonated morphine (orange) are taken from the PMF calculations in Figures 1D, 5. Diffusion through the solvent (ΔG_3_) is assumed to be 0. **(B)** Comparison of the free energy of binding to MOPr directly *via* the aqueous solvent, or indirectly *via* the membrane, where ΔG_indirect_ = ΔG_1_ + ΔG_2_ + ΔG_3_. **(C)** 2D representation of the indirect, lipid binding route, using the same values as **(A)**. Fentanyl (green) has a greater propensity to move into the lipid from the solvent, than morphine (orange).

In order to compare the full binding pathways from solvent to MOPr for fentanyl and morphine, we used the data from the PMF analyses in Figures 1D, 5 to construct free energy landscapes for both ligands in their protonated forms as they interact with MOPr (Figure 6). Figure 6A shows a thermodynamic cycle for each ligand, where ΔG_1_ is free energy of transfer between the receptor and the membrane, as measured in Figures 5C,D, ΔG_2_ is between the membrane and solvent, as per Figure 1D, ΔG_3_ is the energy of moving in the solvent (assumed to be 0 kJ mol^−1^) and ΔG_direct_ represents the aqueous pathway from solvent to orthosteric binding site in the receptor explored in Figures 5A,B. From this, we can state that:

$$\Delta {\text{G}}_{\mathrm{direct}}=\Delta {\text{G}}_{1}+\Delta {\text{G}}_{2}+\Delta {\text{G}}_{3}=\Delta {\text{G}}_{1}+\Delta {\text{G}}_{2}$$

As can be seen in Figure 6B, this indeed holds up, and the energies we have obtained here agree whether measured for the direct binding route or the indirect route, *via* the membrane. Importantly, whilst the overall binding energy for each ligand is very similar, the primary difference is the increased preference of fentanyl to partition into the lipid membrane (Figure 6C) where it can access the lipophilic access route. This suggests that fentanyl may favour this indirect, lipid access route, whereas morphine, which does not penetrate into the lipid, favours the “canonical” pathway, binding directly from the aqueous solvent.

### Mutagenesis of the TM6/TM7 Lipid Access Route

**FIGURE 7:**
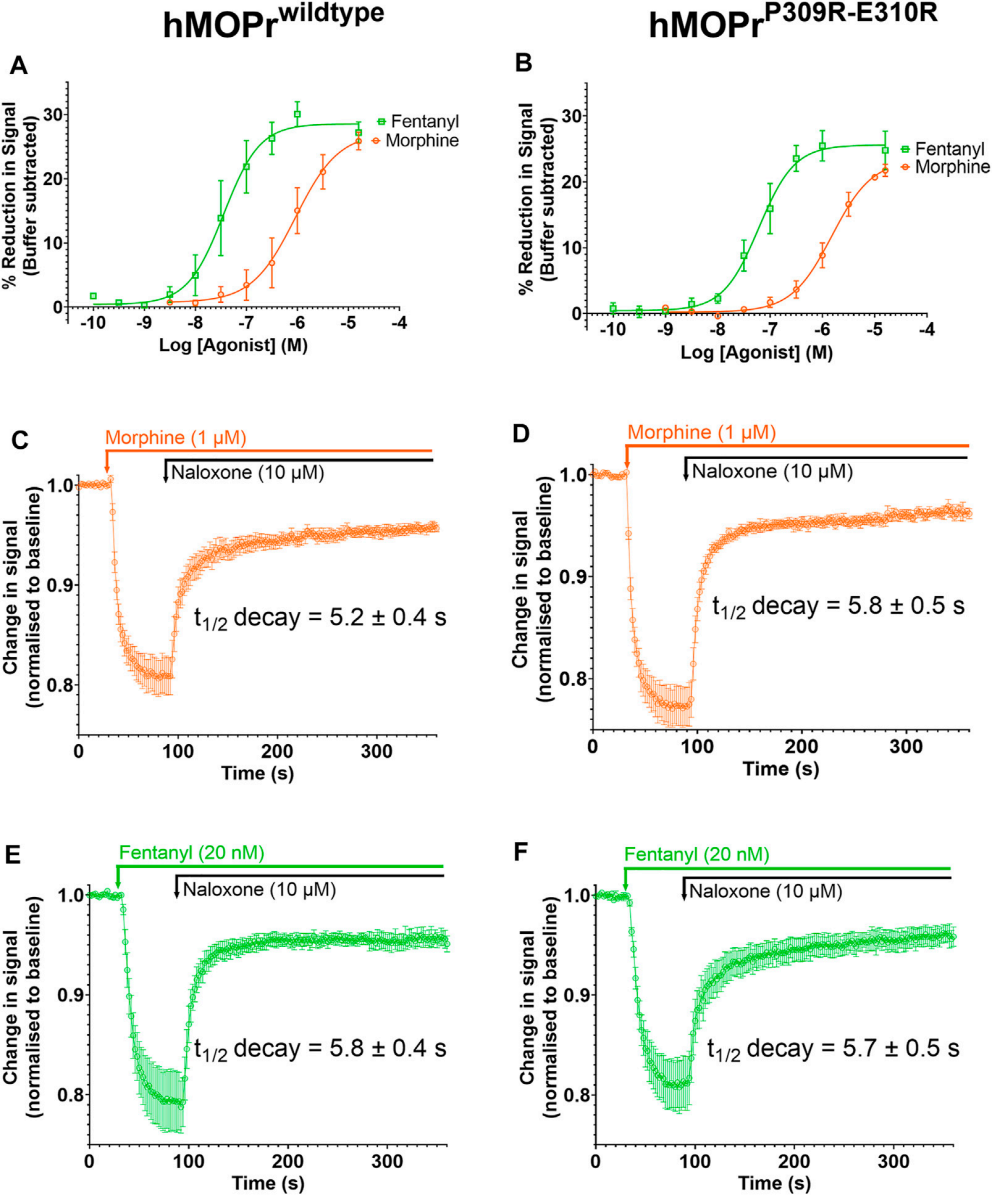
Opioid-induced signaling in AtT20 cells expressing WT-MOPr or MOPr^P309R−E310R^. **(A,B)**. Concentration response curves for fentanyl (green) and morphine (orange) in a membrane potential assay of AtT20 cells expressing **(A)** WT-MOPr and **(B)** the MOPr^P309R−E310R^ double mutant. **(C,D)**. Naloxone reversal of the morphine (1 µM) response in **(C)** WT-MOPr and **(D)** MOPr^P309R−E310R^ double mutant expressing cells. **(E,F)** Naloxone reversal of the fentanyl (20 nM) response in **(E)** WT-MOPr and **(F)** MOPr^P309R−E310R^ double mutant expressing cells. All data are shown as mean ± SEM, N = 5–6.

We next sought to mutate residues forming the TM6/TM7 interface to determine how this would affect fentanyl pharmacology. As highlighted above, our MD simulations of fentanyl binding suggested that the smaller hydrophobic side chains around this site were important in allowing formation of the gap through which fentanyl penetrates. Comparison of the residues in TM6, TM7 and ECL3 in the MOPr with those of the δ-opioid receptor (DOPr) revealed that the proline of MOPr (P309) is replaced with two arginine residues (R291 and R292) in the DOPr. Fentanyl has approximately 400-fold lower potency at the DOPr, compared to the MOPr (62). We therefore hypothesized that these positively charged and bulky arginine side chains might impede fentanyl binding by both repulsion of the protonated nitrogen and steric hinderance. We therefore generated a MOPr double mutant, MOPr^P309R−E310R^, and stably expressed it in AtT20 cells to use in a fluorescence-based assay of MOPr coupling to GIRK channel activation to produce membrane hyperpolarization (59). Cells were treated with a membrane potential-sensitive dye and then with opioid agonists (Figure 7). Activation of MOPr was measured as a change in fluorescence (59).

Figures 7A and B show concentration-response curves for morphine and fentanyl in the WT-MOPr and MOPr^P309R−E310R^ expressing cells. The mutations did not alter the relative potencies of morphine and fentanyl to activate MOPr. Next, we determined if the mutations would alter the apparent off-rate of agonist binding to MOPr in the presence of a high concentration of naloxone (10 mM; Figures 7C–F). The MOPr^P309R−E310R^ mutations did not alter the apparent off-rate of fentanyl or morphine compared to WT-MOPr cells. We conclude that replacement of P309 and E310 with arginine does not alter the *in vitro* pharmacology of morphine or fentanyl.

## Discussion

**FIGURE 8:**
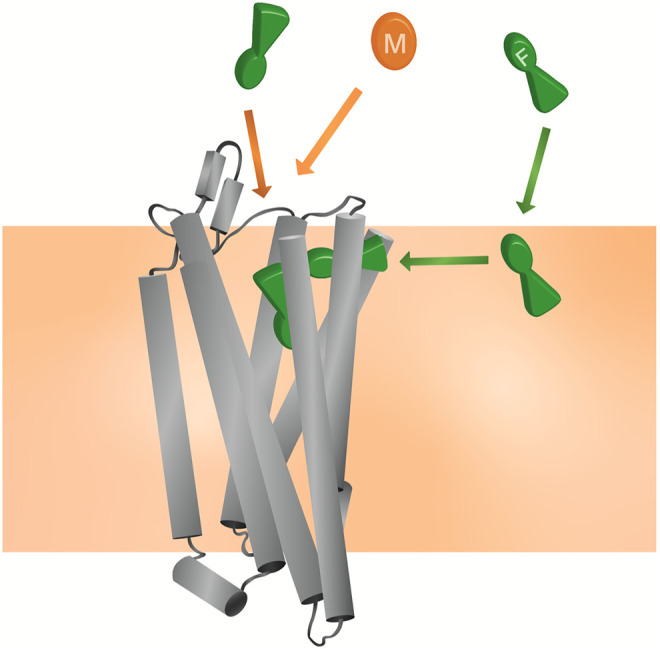
Model for the unique pharmacology of fentanyls at the MOPr In competition with a morphinan ligand (such as morphine or naloxone), fentanyl (green) can access the orthosteric pocket *via* two binding routes; the canonical aqueous pathway and by the novel lipid pathway. In contrast, the morphinan ligand (orange) only has access to one binding route.

Here, we applied CG MD simulations to study the interactions of both fentanyl and morphine with the MOPr and the lipid bilayer. Using a combination of unbiased MD simulations and free energy calculations, we observed that *in silico* fentanyl exhibited a marked preference to partition into the lipid, congregate around the receptor TMDs, and potentially access the MOPr orthosteric site *via* a novel binding route through the lipid membrane and MOPr TMDs (Figure 8). Whereas, morphine did not concentrate around the MOPr, nor did it penetrate the bilayer sufficiently to access the lipid binding route. Instead, morphine accessed the orthosteric pocket by diffusing directly from the aqueous solvent and an extracellular vestibule site. Free energy calculations showed that whilst fentanyl can also bind to the MOPr *via* the canonical aqueous route, fentanyl’s high lipid solubility allows it to partition into the membrane where it can gain access to the lipid binding route.

Using electrophysiological recordings from LC neurons, we show that, unlike morphine, fentanyl can re-assert its action after washout of fentanyl and the antagonist naloxone from the extracellular space. As has been previously shown for β2-adrenoceptor agonists, this phenomenon can be explained by the “microkinetic model” (63), whereby fentanyl accumulates in the lipid where it is unable to be washed out and can then re-bind to the MOPr. This re-binding could either occur *via* the canonical aqueous route, requiring fentanyl to first partition back out of the lipid, or *via* the novel lipophilic route described by our MD simulations. We attempted to block the lipid access pathway by mutating residues in the TM6/7 helical binding route. We hypothesised that effective blockade of the lipid access route would alter the relative potency and dissociation rate of fentanyl, compared to morphine, due to fentanyl only having access to the aqueous binding route in the mutant MOPr. However, our cell signaling experiments with the MOPr^P309R−E310R^ mutant did not show any appreciable difference from WT-MOPr. This does not preclude the possibility that fentanyl binds *via* this lipophilic route, but does suggest that the small hydrophobic P309 sidechain and negatively charged E310 sidechain are not essential for fentanyl to access the lipid pathway. It remains to be determined whether mutation of other residues within the TM6/7 interface would alter fentanyl pharmacology.

Due to the reduced resolution of the CG MD employed in this study, the two ligands represent multiple “fentanyl” or “morphinan” molecules. It is likely that other fentanyls with high lipophilicity could also exhibit membrane partitioning and lipid phase binding to the MOPr, for instance carfentanil, sufentanil and ohmefentanyl. The size of the putative fentanyl-induced gap between TM6 and 7 would suggest that fentanyl’s ability to bind *via* the lipid is a property of both its high lipophilicity and the elongated, flexible structure. Morphine, which is less lipid soluble, would not penetrate into the lipid far enough to access the gap, and is therefore unlikely to favour this binding pathway.

A lipid phase binding route has been proposed for other GPCRs; notably rhodopsin and the CB2 cannabinoid, sphingosine-1-phosphate, PAR1 and P2Y1 receptors (28-30, 64, 65), though not so far for the MOPr which has evolved to recognise non-lipophilic peptide ligands. 2-Arachidonoylglycerol and vorapaxar are reported to access the orthosteric pocket *via* the TM6/7 interfaces of the CB2 and PAR1 receptors, respectively (28, 29). Particularly, in simulations of vorapaxar unbinding from the PAR1 receptor, the ligand also exits *via* a gap formed by TM6/7 and ECL3 (29). Similar to the putative lipid access route in MOPr, this gap is lined by small hydrophobic residues and an aromatic residue in position 7.35 (tryptophan in MOPr, tyrosine in PAR1). In the CB2 receptor, the entry gap is further towards the intracellular side of TM6 and 7 (28).

Could this novel mechanism of interaction with the lipid membrane and with MOPr explain the anomalous pharmacology of fentanyl (12)?

Firstly, by concentrating fentanyl in the bilayer, the apparent concentration around the receptor is markedly increased, as the membrane acts as a reservoir. This high local concentration increases the likelihood of receptor association; either *via* the putative lipid access pathway and/or by enhancing the fentanyl concentration in the extracellular space near the MOPr. Therefore, whilst morphine and fentanyl have very similar binding energies for MOPr, the actual likelihood of fentanyl binding would be far higher, and this might well explain the increased potency of fentanyl over morphine, particularly in cells where a complete, intact cell membrane is present.

Secondly, once fentanyl has partitioned into the bilayer it will switch from 3D diffusion in the solvent to 2D, lateral diffusion in the membrane (66). This reduction in dimensionality results in fentanyl having a greater chance of finding the receptor target, compared to morphinan ligands exhibiting 3D diffusion in the aqueous phase. Similarly, the membrane may also serve to organise the fentanyl molecules at a depth and orientation which favours binding through the TM6/7 interface (67).

Our identification of a potential TM6/7 interface on the outside of the MOPr helices also invites the possibility that fentanyl exhibits “exosite” re-binding, as described by Vauquelin and Charlton (68). Unlike morphinan ligands which bind and unbind *via* the aqueous phase, fentanyl is not free to diffuse away from MOPr and instead binds to the “exosite” TM6/7 interface. From here, fentanyl could then rapidly and efficiently rebind to the orthosteric site.

The mechanisms outlined here may also explain the poor reversibility of fentanyls by the morphinan antagonist naloxone. Naloxone has similar lipid solubility to morphine and is therefore unlikely to concentrate in the bilayer or access the lipid phase binding route (Figure 6). It would therefore only compete with fentanyl for binding *via* the aqueous route, not the lipophilic route. Whilst naloxone can still compete with fentanyl to occupy the orthosteric pocket, fentanyl could remain bound to the TM6/7 exosite and thus rapidly rebind to the orthosteric site once naloxone has dissociated. A similar phenomenon has been demonstrated for the lipophilic β2 adrenoceptor agonist, salmeterol, where the ligand may be retained in the lipid membrane allowing reassertion of its agonist effects after wash-out (69, 70).

Whilst there are advantages to using a CG model to interrogate ligand-lipid interactions, it is important to acknowledge some caveats. Firstly, whilst this manuscript was under review, an updated version of the Martini force field (Martini 3.0) was published (71). This newer force field represents an improvement on the Martini 2.2 version used here, particularly in regard to lipid and water interactions and protein flexibility (71). However, unlike Martini 2.2, Martini 3.0 does not include cholesterol, an important component of the membrane and a potential modulator of opioid action (72). Secondly, as the binding pocket of the MOPr is narrow and the CG water beads are relatively large, the binding pocket was not hydrated during our simulations. Crystal structures of the MOPr have detected water molecules within the orthosteric pocket which engage in interactions with the ligand and form polar networks (73). The role of water within the MOPr pore is likely to be important for opioid ligand binding, and this is unable to be captured by the CG model. Similarly, our CG model is unable to include a sodium ion in the allosteric pocket below the orthosteric site. However, atomistic MD simulations have shown that the presence of sodium in this site only marginally alters the binding pose of opioids (44), and therefore its absence in our CG MOPr is unlikely to affect the binding pathways we observe. Finally, due to the smoothed energy landscape caused by using CG beads, the binding energies estimated here should be taken as a relative comparison between different binding modes, rather an absolute binding energies, as they tend to underestimate the energy barriers between the bound and unbound state (42). Future work incorporating atomistic simulations might help address some of these areas.

Fentanyls are driving the current opioid overdose epidemic in the United States (74). Fentanyl’s rapid onset and high potency are compounded by poor naloxone-reversibility, making the risk of fentanyl overdose high. Only by understanding fully how fentanyl interacts with and activates MOPr will we be able to develop better antagonists. We have recently shown that the more lipophilic antagonist diprenorphine is better able to antagonize the effects of fentanyl, compared to naloxone (10). This might suggest that diprenorphine can at least concentrate in the lipid membrane, and potentially also access the entry point in the TMDs to block fentanyl access. Whilst elucidating how diprenorphine and other lipophilic ligands interact with the MOPr requires further study, the development of lipophilic MOPr antagonists may prove beneficial in combatting fentanyl overdose.

## Data Availability

The raw data supporting the conclusion of this article will be made available by the authors, without undue reservation.
