# Supplementary material for: Interaction With the Lipid Membrane Influences Fentanyl Pharmacology
Source: Adv Drug Alcohol Res. 2022 Mar 21;2:10280. doi: 10.3389/adar.2022.10280 (PMC7613138; doi:10.3389/adar.2022.10280)
Supplement: Supplementary file 2 [file datasheet1.pdf]

Supplementary Methods

In the electrophysiology experiments, the observed reassertion of the fentanyl-evoked current upon wash-out could potentially be an artefact due to leaching of fentanyl that had adhered to the tubing of the perfusion system. To exclude this possibility we perfused fentanyl (100 nM) -containing aCSF through the in-flow tubing of the perfusion system before the system was flushed with air and subsequently perfused with aCSF. This perfusate was collected and then applied to rat LC slices. No GIRK current was observed in LC neurones in response to application of this perfusate (data not shown, N = 3) confirming that the reassertion of the fentanyl-evoked current upon wash-out was not due to fentanyl leaching from the tubing.

Ligand (6 copies)	n	Simulation time (μs)	Ligand at the TM6/7 interface?	Orthosteric site binding?
Fentanyl (protonated)	6	5; 5; 5; 5; 5; 5	Yes; Yes; Yes; No; Yes; Yes	No; No; Yes; No; No; No
Fentanyl (neutral)	6	5; 5; 5; 5; 5; 5	Yes; Yes; No; Yes; No; Yes	No; No; No; No; No; No
Morphine (protonated)	3	1; 5; 1	No; No; No	No; Yes; No
Morphine (neutral)	3	1; 1; 1	No; No; No	No; No; No

Supplementary Table S1. Long-timescale, independent CG simulations for each opioid ligand

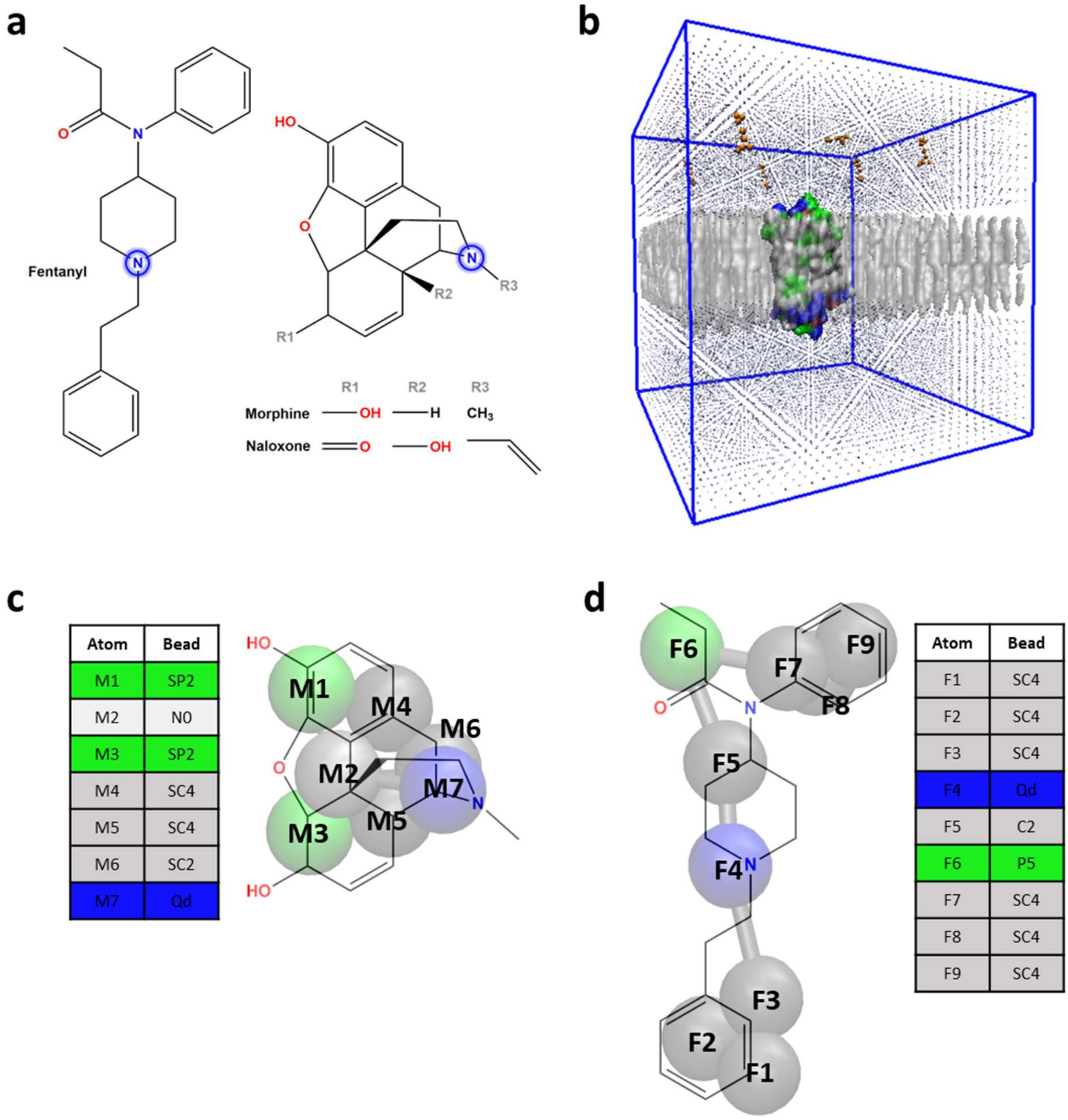

Atom	Bead
M1	SP2
M2	N0
M3	SP2
M4	SC4
M5	SC4
M6	SC2
M7	Qd

Atom	Bead
F1	SC4
F2	SC4
F3	SC4
F4	Qd
F5	C2
F6	P5
F7	SC4
F8	SC4
F9	SC4

Supplementary Figure S1. Ligand parameterization and system set up
(a) Elongated structure of fentanyl compared to the rigid ring structures of morphine and naloxone. The protonatable amine in both molecules is highlighted. **(b)** The systems were set up with CG MOPr embedded in a POPE, POPC, cholesterol membrane (grey), solvated in water and ion beads (blue) and 6 molecules of either fentanyl or morphine (orange) randomly placed in the solvent. **(c)** Morphine was parameterized for the Martini forcefield using 7 Martini beads. **(d)** Fentanyl was parameterized for the Martini forcefield using 9 Martini beads. For simulations of neutral (unprotonated) ligands the Qd beads were replaced with Nd beads. For explanation of MARTINI bead types, see *Marrink et al 2007.*

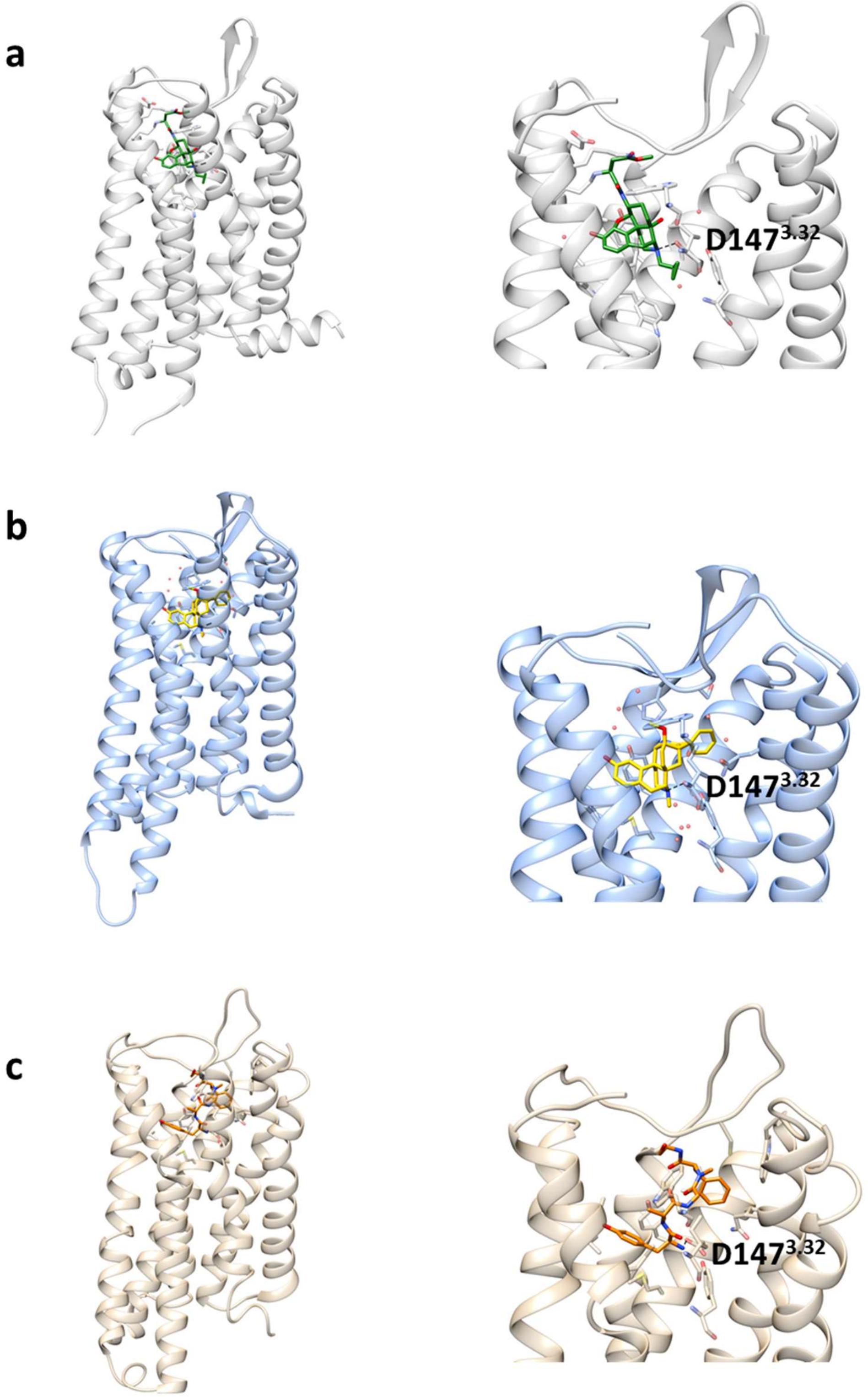

Supplementary Figure S2. Structures of the MOPr
(a) X-ray crystal structure of MOPr (grey) bound to the antagonist β-FNA (green). PDB 4DKL *(Manglik et al. 2012)*. **(b)** X-ray crystal structure of MOPr (blue) bound to the agonist BU72 (yellow). PDB 5C1M *(Huang et al. 2015)*. **(c)** Cryo-EM structure of MOPr (tan) bound to the peptide agonist DAMGO (orange). PDB 6DDF *(Koehl et al 2018)*. In each case, residues forming the binding pocket are displayed as sticks and the key amine-D147$^{3.32}$ interaction is indicated with a dashed line.

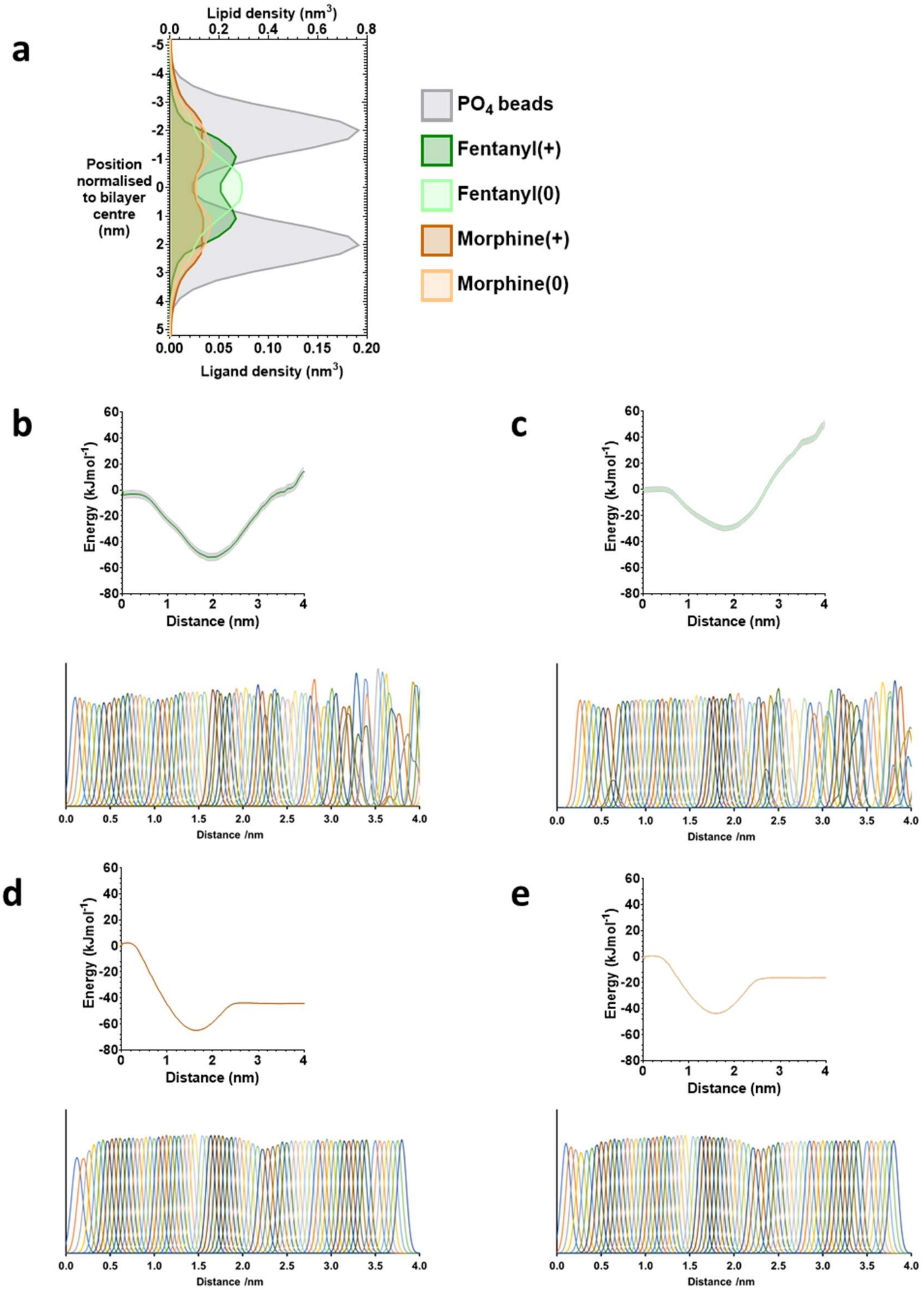

Supplementary Figure S3. Free energy calculations for ligand solvent/membrane partitioning

(a) Density plots showing the average position of protonated fentanyl (dark green), neutral fentanyl (light green), protonated morphine (dark orange) and neutral morphine (light orange), in relation to the phosphate beads of the lipid membrane (grey). **(b-d)** Full PMF profiles along the reaction coordinate for the umbrella sampling simulations of ligands partitioning between the centre of the lipid bilayer (0 nm) and the bulk aqueous solvent (4 nm): **(b)** protonated fentanyl, **(c)** neutral fentanyl, **(d)** protonated morphine and **E.** neutral morphine. The histograms alongside each PMF profile indicate the umbrella sampling windows fully captured the entire reaction coordinate.

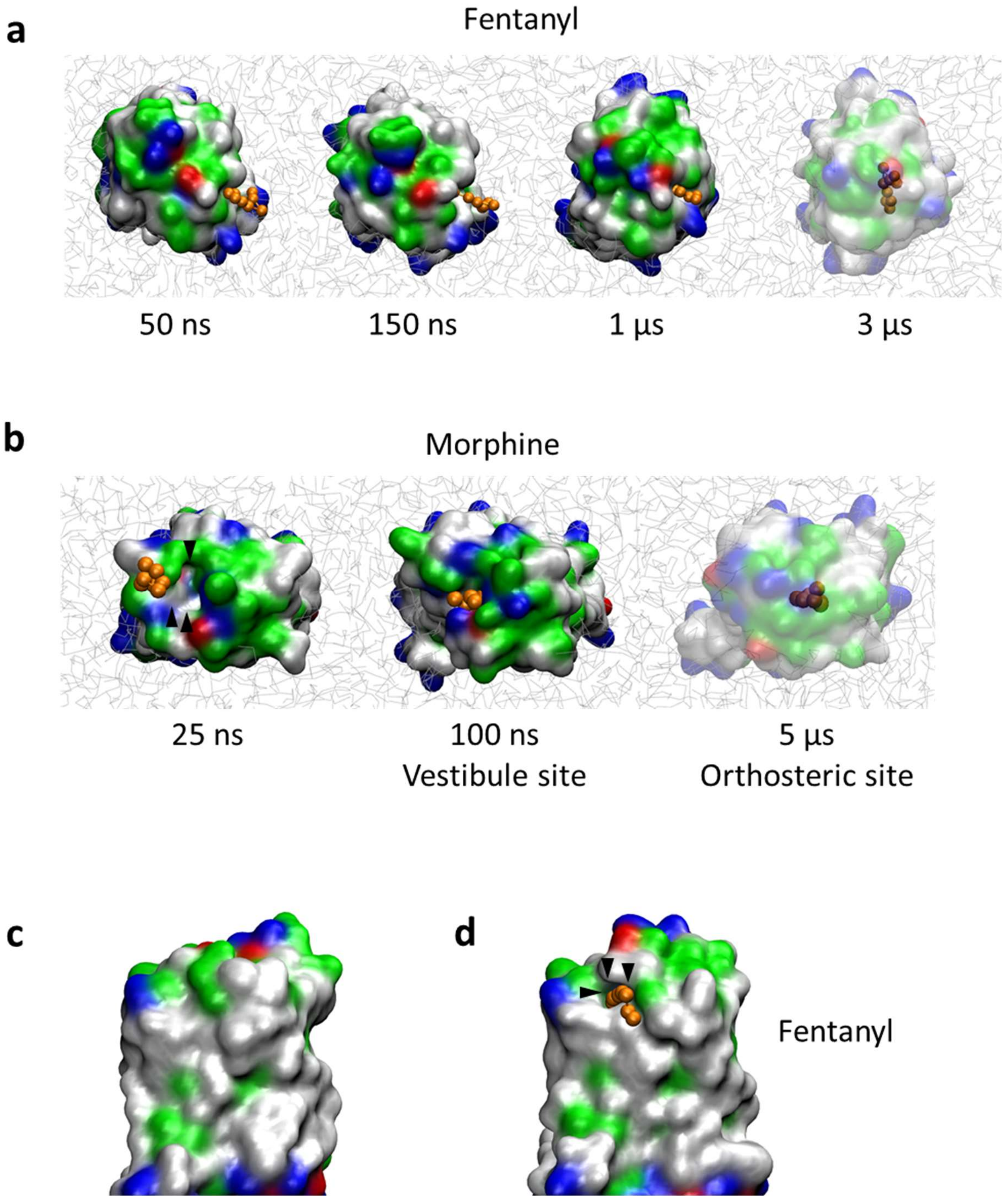

Supplementary Figure S4. Opioid ligand binding to the MOPr

(a) Fentanyl (orange) binding via the lipid membrane, with the MOPr viewed from the extracellular side of the membrane. **(b)** Morphine (orange) binding via the extracellular vestibule (arrows), with MOPr viewed from the extracellular side of the membrane. **(c)** Surface representation of the TM6/7 interface in the absence of fentanyl. **(d)** Fentanyl induces formation of a gap between TM6/7 (arrows) through which the ligand can bind. The protein is coloured according to residue properties (hydrophobic; grey, polar; green, acidic; red, basic; blue).

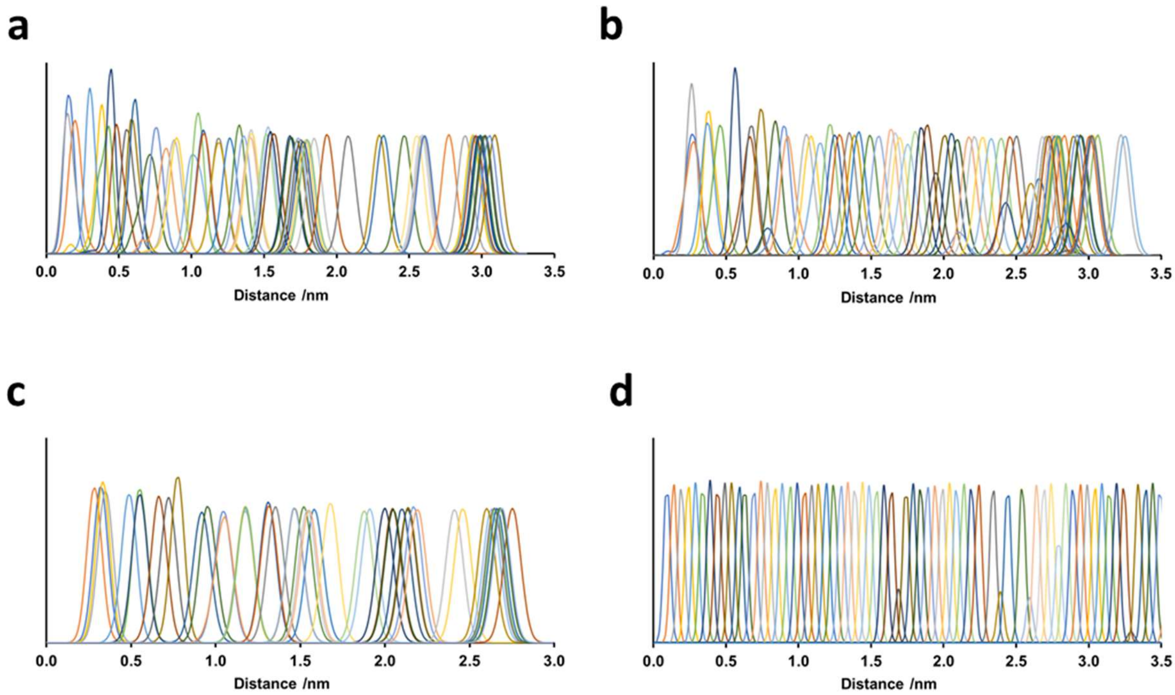

Supplementary Figure S5. Free energy calculations for ligand binding pathways
(a) Histograms along the reaction coordinate for the umbrella sampling simulations of morphine binding via the aqueous pathway. **(b)** Histograms along the reaction coordinate for the umbrella sampling simulations of fentanyl binding via the aqueous pathway. **(c)** Histograms along the reaction coordinate for the umbrella sampling simulations of morphine binding via the lipid pathway. **(d)** Histograms along the reaction coordinate for the umbrella sampling simulations of fentanyl binding via the lipid pathway.

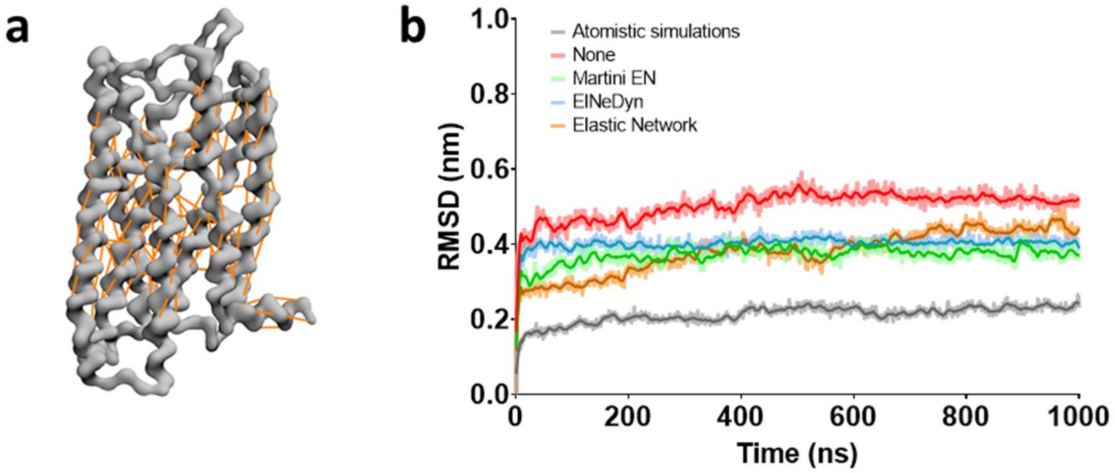

Supplementary Figure S6. Elastic network
(a) Depiction of the elastic network applied to the MOPr model. Elastic bonds are shown in orange. MOPr BB helices in grey. **(b)** RMSD calculations of the protein backbone in atomistic simulations (grey), CG simulations with no elastic network (red), the martini elastic network (green), the ElNeDyn network (blue) and our modified elastic network (orange). Data presented as raw data and moving average (bold lines).

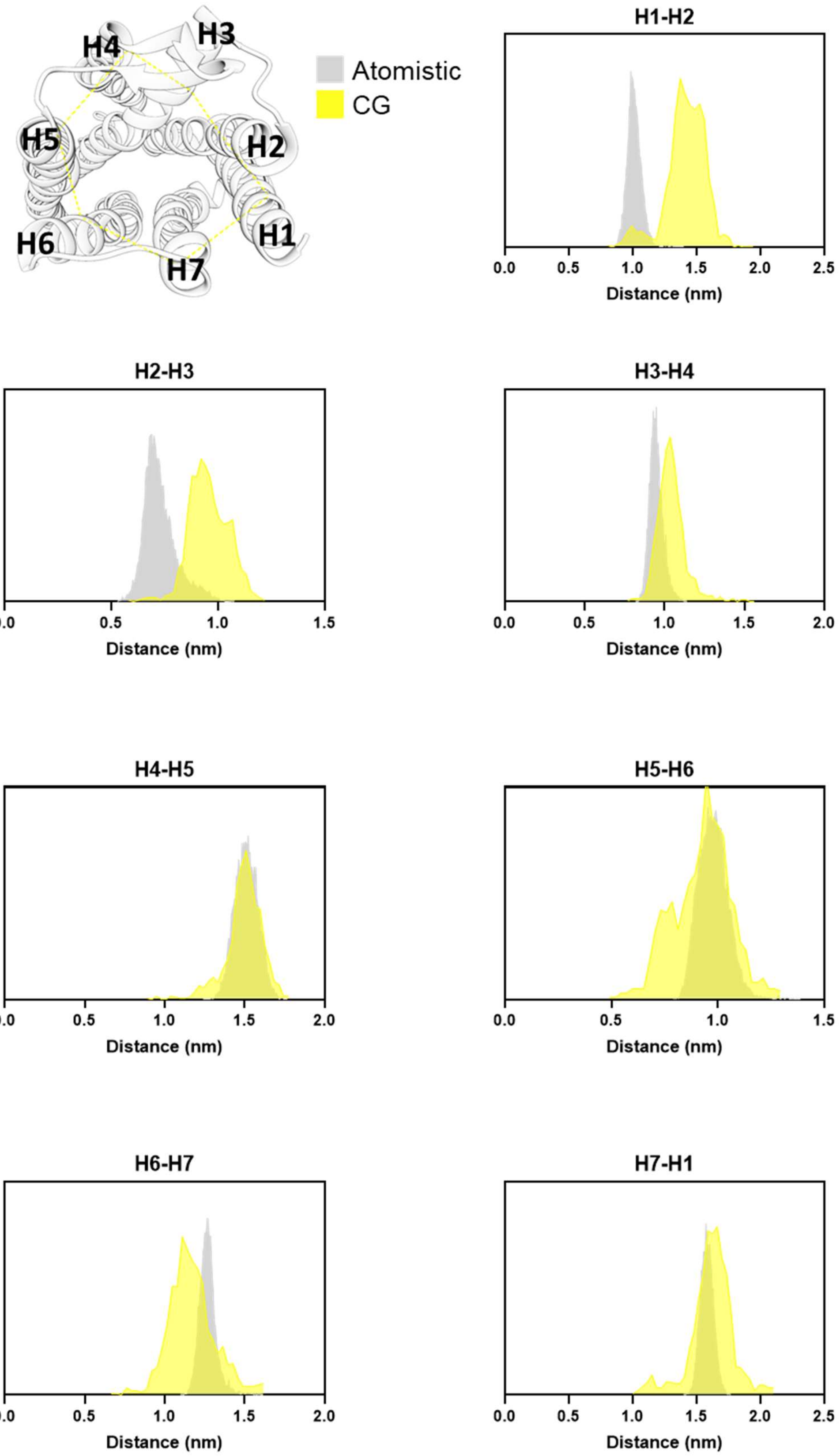

Supplementary Figure S7. Helix distances
Distances between each TM helix in atomistic simulations (grey) and CG simulations (yellow).

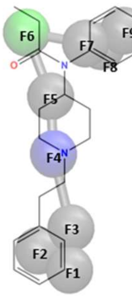

Bond#	Beads
0	1-2
1	1-3
2	2-3
3	3-4
4	4-5
5	5-6
6	6-7
7	7-8
8	7-9
9	8-9

Angle#	Beads
0	1-2-3
1	2-3-1
2	3-1-2
3	2-3-4
4	1-3-4
5	3-4-5
6	4-5-6
7	5-6-7
8	6-7-8
9	7-8-9
10	8-9-7
11	9-7-8
12	6-7-9

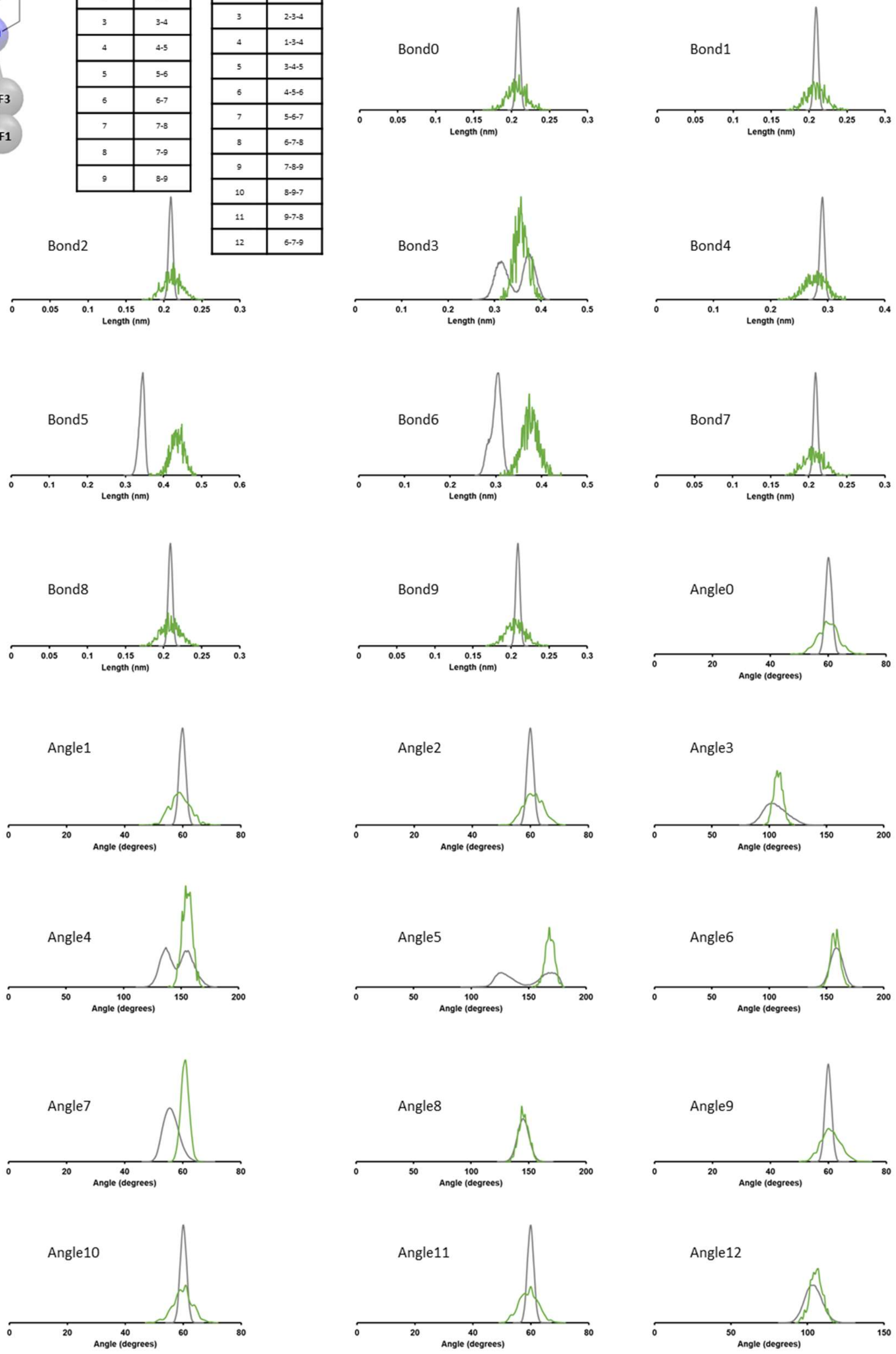

Supplementary Figure S8. Fentanyl geometries

Comparison of the bond lengths and angles in simulations of all-atom (grey) and CG fentanyl (green).

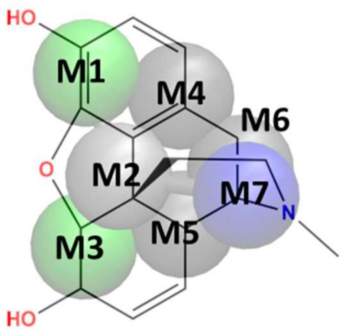

Bond#	Beads
0	1-2
1	1-4
2	2-4
3	2-7
4	2-5
5	2-3
6	3-5
7	4-6
8	5-6
9	6-7

Angle#	Beads
0	2-1-4
1	1-4-2
2	4-2-1
3	1-2-3
4	2-3-5
5	3-5-2
6	5-2-3
7	1-4-6
8	2-4-6
9	4-2-5
10	4-2-7
11	2-5-6
12	4-6-7
13	5-6-7
14	6-7-2
15	5-2-7
16	4-6-5
17	3-5-6

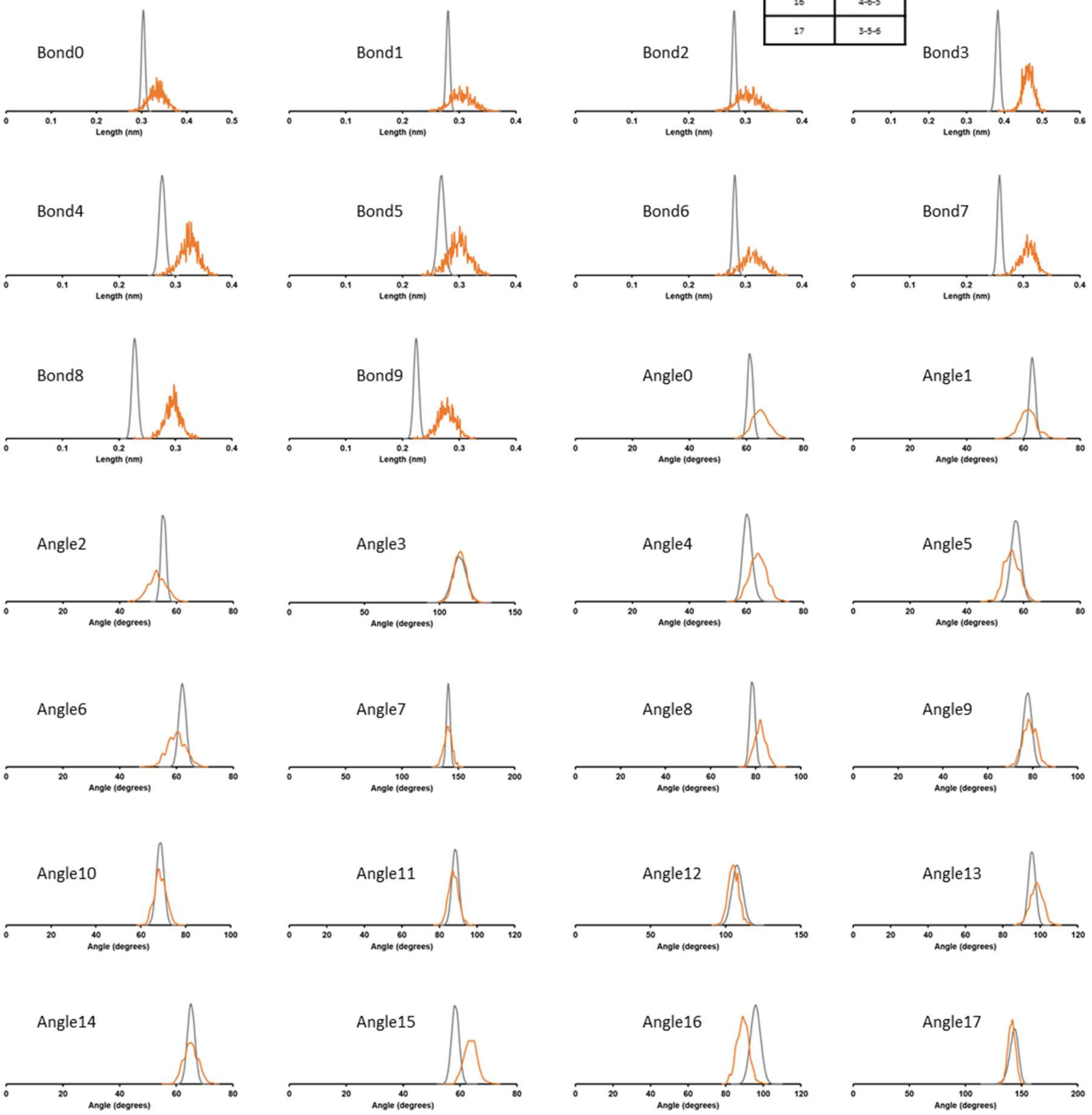

Supplementary Figure S9. Morphine geometries
Comparison of the bond lengths and angles in simulations of all-atom (grey) and CG morphine (orange).

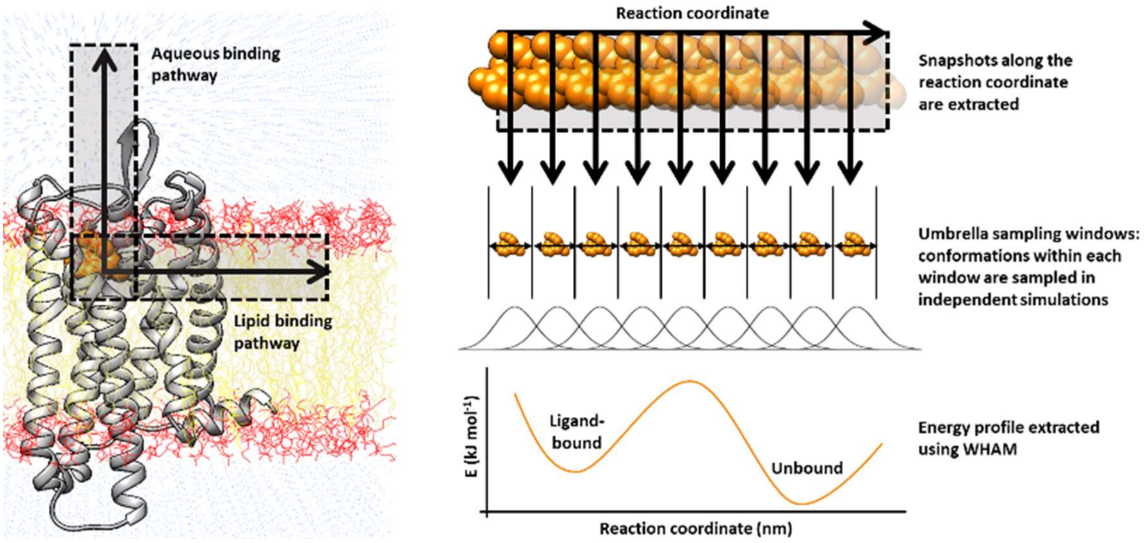

Supplementary Figure S10. Calculating free energy values by steered MD and umbrella sampling
Steered MD uses a pulling force to generate a trajectory from which snapshots can be extracted to seed umbrella sampling simulations. Independent, overlapping umbrella sampling simulations allow efficient sampling of each window along the reaction coordinate. The full energy profile along the reaction coordinate can then be extracted.

Supplementary Movie 1. Fentanyl binding via the lipid bilayer and transmembrane domains

Supplementary Movie 2. Morphine binding via the aqueous solvent and extracellular vestibule
